# Application of Cooling Layer and Thin Thickness Between Coolant and Cavity for Mold Temperature Control and Improving Filling Ability of Thin-Wall Injection Molding Product

**DOI:** 10.3390/polym17192658

**Published:** 2025-09-30

**Authors:** Tran Minh The Uyen, Pham Son Minh, Bui Chan Thanh

**Affiliations:** Faculty of Mechanical Engineering, HCMC University of Technology and Education, Ho Chi Minh City 71307, Vietnam; uyentmt@hcmute.edu.vn (T.M.T.U.); minhps@hcmute.edu.vn (P.S.M.)

**Keywords:** thin-walled mold, cooling layer, heat transfer, mold temperature, injection molding, injection pressure, ANSYS CFX simulation, Moldex3D simulation

## Abstract

Effective thermal management of molds is a governing factor of the quality and stability of the injection molding process. This study introduces and validates an integrated cooling layer within a thin-walled insert mold designed to enhance thermal control and cavity filling performance. A coupled heat transfer simulation model was developed and subsequently calibrated against experimental temperature measurements. To isolate the mold’s intrinsic thermal response, temperatures were measured during distinct heating and cooling cycles, free from the perturbations of polymer melt flow. The validated mold was then installed on a Haitian MA1200 III injection molding machine to conduct molding trials under various injection pressures. A strong correlation was found between the simulation and experimental results, particularly as pressure increased, which significantly improved cavity filling and reduced the deviation between the two methods. The integrated cooling layer was shown to enhance heat dissipation, minimize thermal gradients, and promote a more uniform thermal field. This, in turn, improved filling stability, especially at moderate injection pressures. These findings provide robust quantitative data for simulation model calibration and mold design optimization, highlighting the potential of advanced cooling strategies to significantly enhance injection molding performance.

## 1. Introduction

Plastic injection molding represents one of the most critical manufacturing technologies in contemporary industry, enabling the mass production of high-precision components with significant economic efficiency. Nevertheless, the efficacy and quality of this process are considerably influenced by mold thermal management, wherein the cooling phase constitutes as much as 60–80% of the total cycle time [1]. The optimization of heat transfer within the mold has therefore emerged as a key area of research, aimed at the simultaneous enhancement in surface finish, minimization of part deformation, and reduction in the overall cycle time.

The injection molding of thin-walled parts represents a pivotal direction in modern plastic manufacturing, particularly in the electronics, automotive, and biomedical sectors, where components demand both high dimensional accuracy and light weight. As wall thickness decreases to below 1 mm, the molten polymer encounters significant flow resistance due to a high length-to-thickness (L/t) ratio, leading to risks of short shots or poor surface quality [2]. Under these conditions, high injection pressure and speed are crucial for ensuring complete cavity filling, yet they concurrently elevate residual stresses and thermal deformation. The primary challenge in designing and operating a thin-wall injection molding process, therefore, lies in optimizing processing parameters to balance fillability with the final part’s mechanical integrity [3]. Recent studies indicate a strong interaction effect between mold temperature and injection pressure on the moldability of thin-walled parts. While a high mold temperature reduces melt viscosity, thereby improving flow length and surface finish, high injection pressure ensures the filling of regions with large L/t ratios [4]. However, an improper combination of these factors can lead to warpage, shrinkage, and dimensional instability. Consequently, a successful manufacturing strategy for thin-walled parts not only hinges on material selection and runner design but also necessitates an optimal synergy between thermal and pressure conditions. This underscores the importance of integrating numerical simulations with experimental verification to establish a robust processing window, enabling accurate process prediction and minimizing defects in mass production.

Conventional cooling systems, typically fabricated using straight-drilled channels, result in non-uniform temperature distribution and the formation of hot spots within the mold cavity. This prolongs cooling times, induces warpage, and degrades the quality of injection-molded products. To overcome these limitations, CCC (Conformal Cooling Channel) technology has been developed, enabled by additive manufacturing techniques such as metal 3D printing. CCCs allow the cooling channels to closely follow the contour of the mold cavity, thereby significantly improving heat transfer efficiency, reducing warpage, and shortening the injection molding cycle by 20–40% [5,6,7]. Furthermore, advanced CCC designs incorporate complex geometries, such as TPMSs (Triply Periodic Minimal Surfaces). These structures not only increase the heat exchange surface area but also promote a more uniform temperature distribution, which is particularly beneficial for thin-walled products and geometrically intricate parts [8,9]. This development establishes CCCs as one of the most critical research and application frontiers for optimizing modern injection molding processes. A study on the design and fabrication of CCCs using in situ maraging steel via LPBF (Laser Powder Bed Fusion) reported a cooling time reduction of several seconds and experimentally verified lower warpage compared to straight channels after the conformal geometry was optimized through thermal simulations [10].

RHCM (Rapid Heat Cycle Molding) is an advanced technology developed to overcome the common limitations of conventional injection molding, particularly issues such as weld lines, poor surface aesthetics, and insufficient cavity filling in thin-walled parts. The principle of this method involves rapidly heating the mold surface prior to the filling stage and subsequently cooling it rapidly thereafter. This transient temperature variation enhances polymer melt flowability and extends flow length while simultaneously reducing residual stresses and improving the final product’s surface quality [11,12]. Both experimental and simulation studies have demonstrated that RHCM can significantly eliminate weld lines, improve surface gloss, and increase the bond strength at material joining interfaces. Furthermore, when RHCM is integrated with other solutions, such as CCC, the efficiency of mold temperature control and overall product quality are comprehensively optimized while maintaining a reasonable production cycle time [13,14,15]. This indicates that RHCM is not only beneficial for products demanding high aesthetic quality but also possesses broad potential for application in modern industrial manufacturing.

The concept of a “Cooling Layer” has recently emerged as a potential strategy to enhance thermal control in injection molds. Unlike conventional straight-drilled cooling channel systems, which are prone to non-uniform temperature distribution and the formation of localized hot spots, the Cooling Layer is designed as a thin plate integrating coolant channels directly beneath the cavity surface. This structure significantly shortens the heat transfer path and intensifies the thermal exchange between the mold and the cooling medium [16]. Consequently, the cooling time is reduced, temperature differentials are minimized, and the molded part achieves higher dimensional stability.

Experimental and simulation studies indicate that the Cooling Layer can reduce temperature fluctuations on the mold surface by up to 30% compared to conventional channel layouts while substantially shortening the cooling time [17]. Furthermore, this solution offers high design flexibility, allowing the channel geometry to be optimized to closely follow the cavity contour, achieving an efficacy comparable to CCC but with a simpler manufacturing process. The Cooling Layer can also be combined with inserts made from high-thermal-conductivity materials, such as copper alloys, to further augment heat transfer efficiency while maintaining the mold’s structural integrity [18]. Overall, the Cooling Layer represents a viable industrial solution for enhancing mold temperature control. Owing to its ability to shorten cycle times, mitigate warpage, and ensure product surface quality, this technology is considered an effective alternative or supplement to other advanced cooling strategies like CCCs or RHCM.

In optimizing heat transfer for injection molds, several advanced solutions have been proposed, prominently including CCC, RHCM, and the Cooling Layer concept. Each method presents distinct advantages and limitations, contingent on specific technological requirements and industrial applicability. CCCs, enabled by additive manufacturing, allow cooling channels to closely follow the cavity contour, significantly improving heat transfer efficiency and reducing part warpage. However, the fabrication of CCCs is complex, costly, and often restricted to specialized mold applications [19]. In contrast, RHCM operates on the principle of rapidly heating the mold surface before melt injection and cooling it immediately after. This method can eliminate weld lines, enhance surface gloss, and improve the bond strength at material interfaces. Nevertheless, RHCM is energy-intensive and requires specialized heating equipment, which increases operational costs and limits its widespread adoption [20].

The Cooling Layer is regarded as a balanced solution. Its structure, featuring thin layers of cooling channels positioned near the mold surface, both shortens the heat transfer path—analogous to a thin-walled mold—and distributes temperature uniformly, similar to CCCs but with a simpler manufacturing process. Experimental and simulation studies have demonstrated that the Cooling Layer can reduce cooling time by up to 30% and minimize thermal gradients while maintaining the mold’s mechanical integrity, thereby ensuring its feasibility for industrial production [21]. Therefore, the Cooling Layer stands out as an effective and sustainable solution with broader application potential than CCCs and RHCM in the context of mass production.

Designing molds with thin walls is a key strategy for optimizing heat transfer efficiency in injection molding. By reducing the mold wall thickness, the thermal path from the cavity surface to the cooling channels is shortened, which facilitates rapid heat removal and significantly reduces the cooling time—a phase that typically dominates the injection cycle [22]. Experimental studies have shown that decreasing the mold wall thickness from 20 mm to 8–10 mm can shorten the cooling time by up to 30% while promoting a more uniform temperature distribution across the mold surface [23]. Furthermore, the heat transfer efficiency of thin-walled molds can be enhanced by incorporating inserts made from high-thermal-conductivity materials, such as (Be–Cu) beryllium-copper alloys. Empirical investigations reveal that adding these inserts can improve heat transfer by an additional 8–16% compared to conventional steel molds while maintaining mechanical strength and thermal fatigue resistance [24,25,26]. However, thinning the mold walls also introduces challenges related to service life and residual stress, necessitating a trade-off between cooling efficiency and structural integrity to ensure feasibility in industrial production. Thus, thin-walled molds not only contribute to shorter cycle times but also provide a foundation for integration with other advanced methods, such as Cooling Layers or CCCs, to comprehensively optimize cooling performance and product quality.

In injection molding technology, the synergy between thin-walled mold design and the Cooling Layer solution plays a pivotal role in managing the relationship between mold temperature and injection pressure. As the mold wall is thinned, the thermal path from the cavity surface to the cooling channels shortens, which increases the heat transfer rate but concurrently causes the mold surface temperature to drop rapidly during the filling stage. This temperature decrease leads to higher flow resistance, necessitating greater injection pressure to ensure complete cavity filling, particularly for thin-walled parts with high L/t ratios [27]. A Cooling Layer, with its cooling channels positioned close to the mold surface, can maintain temperature uniformity and mitigate this rapid temperature drop, thereby reducing the injection pressure required to achieve the same flow length [8]. Simulation and experimental studies have demonstrated that when a Cooling Layer is integrated into a thin-walled mold, the interaction between mold temperature and injection pressure becomes pronounced: a higher mold temperature reduces melt viscosity to aid cavity filling, while the Cooling Layer ensures uniform cooling to limit residual stresses and part warpage [28]. Thus, the simultaneous optimization of these two factors not only enhances heat transfer efficiency and surface quality but also establishes an experimental and simulation database for selecting appropriate processing parameters, aimed at improving the stability and efficiency of the entire injection molding cycle.

Although individual optimization strategies such as thin-walled molds, CCCs, or RHCM have proven effective in improving heat transfer and product quality, there remains a limited number of studies that simultaneously integrate these solutions. The novelty of this research is the proposition of a thin-walled insert incorporating a Cooling Layer—a design that synergistically combines the advantages of thin-walled molds (shortened heat transfer path), CCCs (enhanced temperature uniformity), and RHCM (improved surface finish, weld line elimination). Compared to conventional conformal cooling channels, this solution emphasizes industrial feasibility by enhancing cooling performance while maintaining the mold’s mechanical strength and service life. Furthermore, the integration of numerical simulation and experimental validation in this study enables a comprehensive evaluation of the correlation between temperature distribution, injection cycle time, and product quality. This work thereby contributes a new, holistic approach to the comprehensive optimization of the modern injection molding process [29,30,31].

## 2. Materials and Methods

### 2.1. Mold Heating and Cooling System with Thin-Walled Insert and Cooling Layer

**Figure 1:**
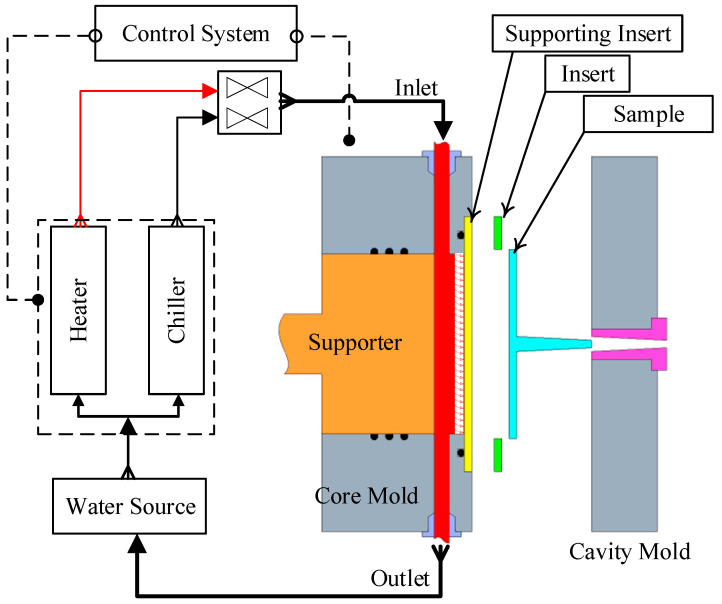
Schematic diagram of the cyclic heating and cooling system using Cooling Layer and thin-walled mold insert.

The mold heating and cooling system was configured as a closed-loop circulation circuit to ensure precise thermal management during the injection molding process (Figure 1). This system comprises a water reservoir, a Haitian water heating unit, a chiller, the mold assembly—featuring thin-walled inserts and a high-conductivity cooling layer—a switching valve, and a central control unit. The injection process is performed by a Haitian MA1200 III machine (Haitian Plastics Machinery Group Co., Ltd., Ningbo, China), while the Haitian water heater delivers fluid at a preset temperature to the mold’s cooling channels, which are positioned proximate to the cavity surface. Real-time process data, including inlet (T_in) and outlet (T_out) temperatures, mold surface temperature (T_mold), flow rate, and pressure, are continuously acquired by integrated sensors and transmitted to the central controller. This controller then computes the deviation from predefined setpoints for both heating and cooling temperatures. A PID (Proportional–Integral–Derivative) control algorithm regulates the switching valve, heating power, and pump flow rate to maintain a uniform and stable mold temperature throughout the cycle. This configuration enables the mold surface temperature to be rapidly elevated during the filling stage to improve melt flowability and enhance surface quality. Subsequently, the temperature is quickly reduced during the cooling stage to accelerate solidification, minimize residual stresses, and mitigate part warpage. The integrated system significantly improves process stability and energy efficiency, thereby demonstrating the potential of combining thin-walled mold inserts with a cooling layer for optimized thermal management in injection molding.

**Figure 2:**
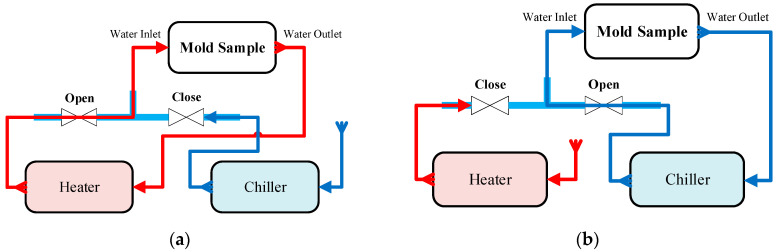
Schematic diagram of the mold temperature control system: (**a**) heating mode using a heater, (**b**) cooling mode using a chiller.

The operational process comprises four main stages: heating (Figure 2a), injection/packing, cooling (Figure 2b), and product ejection. During the heating stage, a control valve directs fluid from the Haitian heating unit, pre-set between 70 °C and 120 °C, into the circuit. This heated water circulates through the cooling layer proximate to the cavity surface, rapidly elevating the mold temperature. The central controller regulates the heating rate (dT/dt) to prevent thermal shock and mitigate potential material damage. Subsequently, the injection and packing stages are initiated by the Haitian MA1200 III machine while the heating circuit is maintained. This approach prevents premature solidification at the gate, enhances melt flowability, and improves cavity filling performance.

Upon completion of filling and packing, the system transitions to the cooling stage. The switching valve re-routes the circuit, delivering chilled water (15–25 °C) from the chiller through the same integrated channel network. A PID-based control algorithm dynamically adjusts the flow rate to optimize the thermal gradient between the heating and cooling phases, thereby maximizing heat transfer efficiency and ensuring a uniform temperature distribution. Finally, product ejection is triggered once the mold surface temperature reaches its preset limit and the thermal deviation across cavity zones falls within a permissible range. Throughout the entire cycle, integrated sensors continuously monitor the system’s water level, pressure, and temperature. This monitoring ensures process safety by preventing thermal overstress, which could otherwise degrade the mechanical durability and fatigue life of the thin-walled mold inserts.

### 2.2. Operating Principle of the Integrated Mold Heating–Cooling System

In injection molding, precise control of the mold temperature is a critical factor governing both product quality and process efficiency. While conventional molding maintains low surface temperatures to shorten the cooling time, this approach exacerbates the risks of premature solidification, weld line formation, and poor surface finish. Conversely, elevating the mold temperature during the filling stage enhances melt flowability and improves the replication of intricate geometries, but at the cost of a prolonged cooling phase. To resolve this trade-off, an integrated heating–cooling circulation system within a single fluid channel offers an innovative solution. This system enables high mold temperatures during filling and rapid cooling post-solidification, thereby optimizing both product quality and cycle time simultaneously.

**Figure 3:**
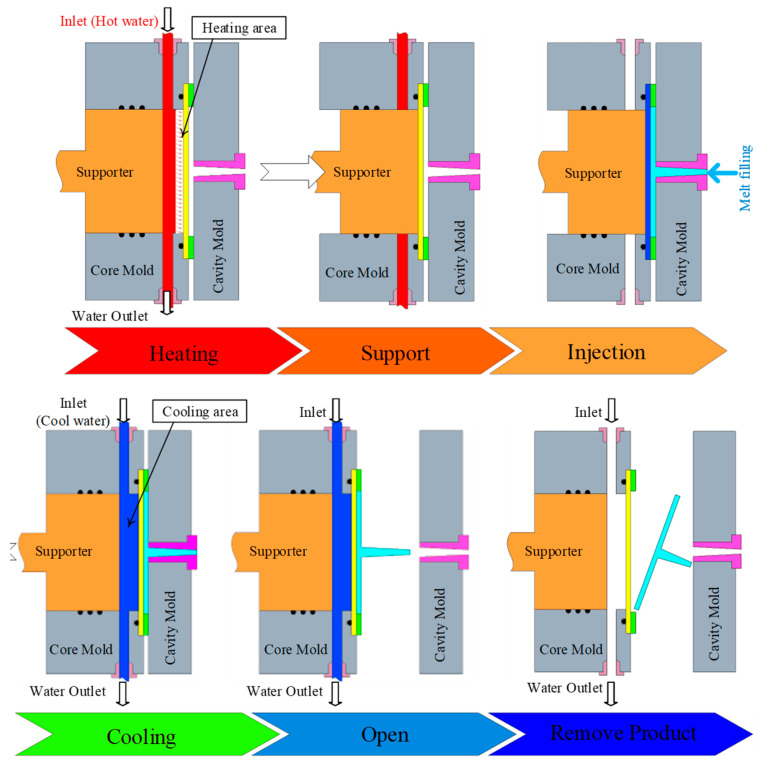
Sequential steps of heating, injection, and cooling in molding process using Cooling Layer and thin-walled mold insert.

Regarding the structural design and operating principle of the proposed system (Figure 3), the thermal transfer fluid layer is thermally insulated from the mold cavity surface by a thin cooling layer. At the onset of the cycle, a heated medium (hot water or high-temperature oil) is circulated through the thermal transfer channel proximate to the cavity surface, allowing the mold surface temperature to rapidly reach the desired setpoint. The thickness of this thin layer plays a decisive role in the heat transfer kinetics: the thinner the wall, the steeper the thermal gradient, leading to faster heating, lower energy losses, and a shorter preparation time for the molding cycle. During the injection stage, the elevated mold temperature enhances melt flowability, reduces apparent viscosity, and mitigates the risk of incomplete filling or premature solidification. Furthermore, maintaining a stable thermal condition at the cavity surface improves surface gloss, reduces weld-line defects, and enhances the dimensional accuracy of the molded parts. Notably, the thin cooling layer simultaneously functions as a fast heat conductor and a temporary thermal barrier, maintaining a high surface temperature throughout the filling process. Immediately after cavity filling, the system automatically switches from the heating to the cooling circuit. This dynamic switching mechanism enables instantaneous thermal quenching, as the stored heat from the mold and polymer melt is rapidly dissipated through the thin cooling layer into the cooling channel. Compared with conventional cooling systems, where channels are positioned farther from the cavity surface, this thin-walled cooling design significantly shortens the heat transfer distance. This results in a faster cooling rate, a shorter cycle time, and improved process repeatability between molding cycles.

Beyond enhancing process performance, this method carries significant scientific implications by demonstrating the optimal integration of two inherently antagonistic heat transfer mechanisms—rapid heating and rapid cooling—within a single mold configuration. By effectively leveraging the thermal properties and inducing turbulent flow of the heat transfer medium, the system maximizes heat transfer efficiency while maintaining a uniform temperature distribution across the entire cavity surface. This capability is particularly critical for thin-walled components, geometrically intricate structures, or products requiring optical-grade surface quality. The cyclic heating–cooling strategy, facilitated by the thin-thickness cooling layer, represents a significant advancement in mold thermal management technology. It not only enhances the quality and dimensional stability of polymer products but also establishes new directions for advanced research. Future investigations should focus on optimizing the cooling-layer channel design, mold material selection, and dynamic thermal control algorithms to further improve industrial production efficiency and product consistency.

The injection molding process integrating a CL (cooling layer) with a thin-cavity mold design consists of six main stages, systematically engineered to optimize thermal conditions and molding cycle efficiency (Figure 3). In the heating stage, hot water within the temperature range of 70–120 °C supplied by the Haitian water heater (power: 12 kW; maximum flow rate: 40 L/min; operating pressure: 0.6 MPa) is circulated into the embedded heating-water cavities located within the core mold. This configuration rapidly raises the mold surface temperature to the desired level, effectively reducing the premature solidification of the polymer melt and facilitating complete cavity filling, which is particularly critical for thin-walled components. Next, the temperature stabilization phase maintains a constant hot-water circulation under controlled pressure and temperature, ensuring a uniform thermal distribution across the entire mold surface, minimizing local thermal gradients, and establishing stable cavity conditions before injection. During the injection stage, the Haitian MA 1200 III injection molding machine (clamping force: 1200 kN; screw diameter: 35 mm; maximum injection pressure: 243 MPa; injection volume: 134 cm^3^) injects molten polypropylene into the cavity at pre-set speeds and pressures. By sustaining a high mold surface temperature, the polymer melt can easily flow into intricate cavity geometries, reducing defects such as weld lines and short shots. Subsequently, the process transitions to the cooling phase, where the supply source switches to cold water within a temperature range of 15–25 °C. The cooling layer enables efficient thermal extraction, facilitating rapid solidification, improved dimensional stability, and enhanced mechanical strength of the molded part. Once the molded component reaches the required temperature, the mold-opening stage separates the core and cavity plates, creating sufficient clearance for part ejection. Finally, the ejection stage utilizes an integrated ejector system to release the molded product without surface deformation or structural damage. Immediately after ejection, the mold closes, and the next injection cycle begins, ensuring continuous operation and high production efficiency.

### 2.3. Design Setup of the Experimental Mold System

**Figure 4:**
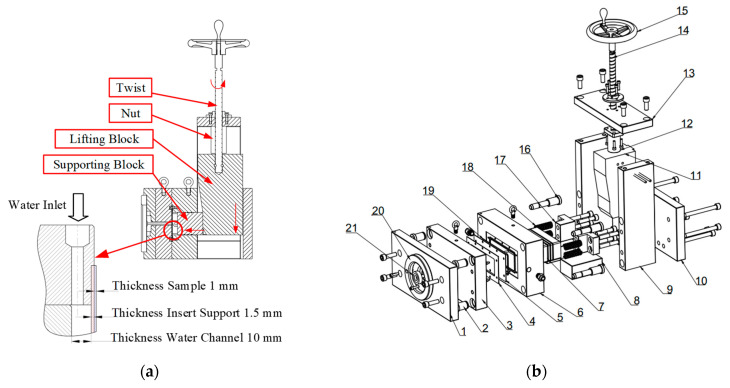
Mold structure: (**a**) Cross-sectional diagram of the mold insert and cooling channel dimensions; (**b**) exploded view showing individual components.

The operating principle of the mold assembly (Figure 4) relies on the coordinated motion of the lead screw and sliding components to ensure precise cavity filling and effective thermal control. Clockwise rotation of the lead screw (14) causes it to translate downward, actuating the movable sliding block (11). This action, in turn, propels the supporting block forward, which then advances the main sliding block (7) toward the insert plate (5). Once the supporting block engages the insert plate, the molten polymer is injected into the cavity under controlled pressure. After the filling stage is completed, the lead screw retracts, creating a clearance between the insert plate and the supporting block that allows cooling water to circulate through the cooling channels. This contact mechanism is critical, as it prevents deformation and local buckling of the insert plate under the high thermal and mechanical loads of the molding process.

The mold assembly utilized in this study (Figure 4) is composed of two primary sections: the cavity side and the core side, with an exploded view presented in Figure 4b. The components, numbered 1–21, define the mold structure; each part serves a specific functional role to ensure dimensional precision and process stability. The cavity side comprises the cavity clamping plate (1), guide pins (2), a locating ring (20), a sprue bushing (21), and the cavity plate (3), which defines the external geometry of the molded product. The core side includes a 1 mm-thick insert sample plate (4), a 1.5 mm-thick insert support plate (5), a rubber seal (19) mounted on the core plate (6), and a spacer block (9) that provides clearance for the ejector system. The cooling system features a 10 mm-thick cooling channel integrated into the supporting block (7), which forms the cooling cavity for water circulation. This design promotes uniform heat dissipation, thereby improving thermal stability and maintaining surface quality.

### 2.4. Numerical Simulation Model

**Figure 5:**
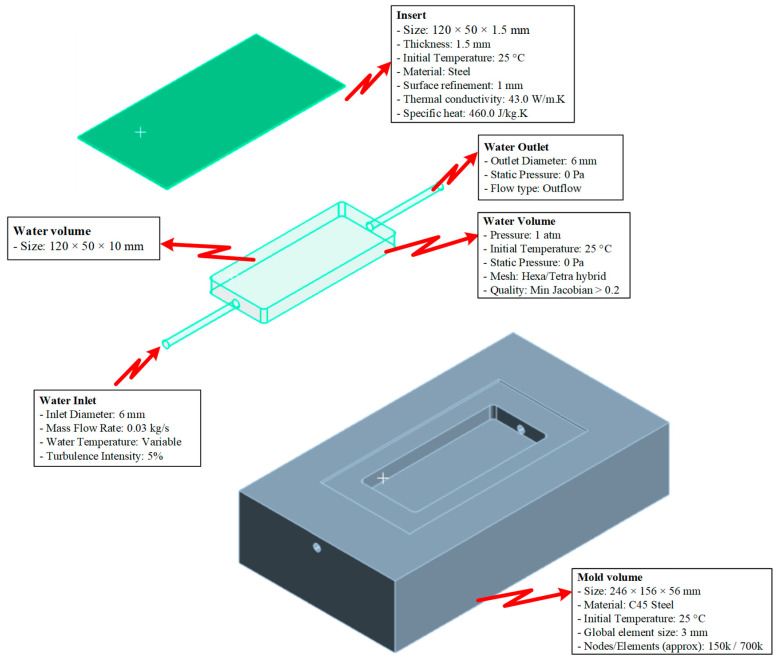
Simulation model.

The simulation model (Figure 5) was developed using ANSYS 2024 R2 to analyze the heat transfer characteristics and fluid flow behavior during the mold cooling process. The model consists of four main components: the insert, mold volume, cooling layer channels, and the coolant volume. The insert has dimensions of 120 × 50 × 1.5 mm and is manufactured from steel with a thickness of 1.5 mm and a 1 mm precision-machined surface. The thermal properties assigned include a thermal conductivity of 50.0 W/m·K and a specific heat capacity of 4600 J/kg·K. The mold volume, sized 246 × 156 × 56 mm, is made of C45 steel and initialized at a uniform temperature of 25 °C. For accurate resolution, the computational domain is discretized using an average element size of 2 mm, resulting in approximately 700 k elements and 150 k nodes. The cooling channel system is designed with a diameter of 6 mm, comprising one inlet and one outlet. Cooling water enters the system at a mass flow rate of 0.03 kg/s, while the inlet temperature varies depending on the simulation scenarios, and a turbulence intensity of 5% is applied. The cooling layer volume, measuring 120 × 50 × 10 mm, is modeled to represent the heat exchange region adjacent to the cavity surface. At the outlet boundary, a static pressure condition of 0 Pa and outflow configuration are imposed to stabilize the flow field. The computational grid is generated using a hybrid Hexa/Tetra mesh strategy, ensuring element quality with a minimum Jacobian greater than 0.2 to maintain solution accuracy. This model enables a detailed evaluation of the heat exchange mechanisms between the insert, mold, and cooling system while providing a robust framework for optimizing design parameters to enhance cooling performance and thermal management efficiency in injection molding processes.

**Figure 6:**
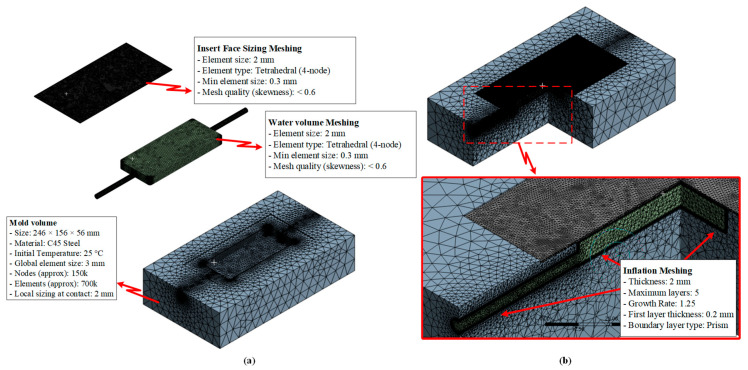
Meshing strategy of the simulation model: (**a**) finite element meshing of different model domains including insert, water volume, and mold volume; and (**b**) inflation meshing method applied at the contact interfaces to improve near-wall heat transfer resolution.

Computational model, meshing parameters, and boundary conditions used for simulating the mold cooling process in injection molding (Figure 6) were established based on the fundamentals of heat transfer and computational fluid dynamics (CFD) to accurately predict heat exchange between the molded product, mold body, and the integrated cooling layer system. The computational domain includes the complete mold geometry, inserts, and cooling channels, enabling the model to simultaneously resolve the three dominant heat transfer mechanisms: (i) solid conduction within mold and insert materials, (ii) forced convection within the cooling water layer, and (iii) thermal contact conduction between the product and the mold cavity surface. An average mesh size of 2 mm was adopted, which has been demonstrated in previous studies [32,33] to be sufficient for capturing temperature and velocity variations in regions with moderate gradients. For thermally sensitive areas, such as the product surface, insert interfaces, and regions adjacent to the cooling layer, local mesh refinement was applied down to 0.3 mm to accurately resolve the formation and evolution of the thermal and hydrodynamic boundary layers. The element quality was maintained with a skewness index below 0.6 to ensure well-shaped elements at intersections, reduce interpolation errors, and improve the convergence stability when solving the coupled Navier–Stokes and energy equations [34]. To capture detailed flow and thermal gradients near cavity walls and sharp corners, an inflation layer was applied, configured with five prism layers, a total thickness of 2 mm, a first-layer thickness of 0.2 mm, and a growth rate of 1.25. At the inlet, boundary conditions were specified with a mass flow rate of 0.03 kg/s, a cooling water temperature of 25 °C, and a turbulence intensity of 5%, ensuring a fully developed turbulent regime (Re greater than 4000). To accurately model this flow, the Shear Stress Transport k-ω turbulence model was employed. This model was selected for its high accuracy in resolving the viscous sublayer and predicting heat transfer at the channel walls. This turbulent condition is critical as it significantly reduces the thermal boundary layer thickness and enhances convective heat transfer at the fluid-solid [35,36]. The outlet was set to an outflow condition with an absolute static pressure of 0 Pa, ensuring smooth discharge and avoiding backpressure effects that could distort the pressure and velocity distributions. The mold body was modeled using C45 steel with a thermal conductivity of approximately 50 W/m·K, while the inserts were modeled as 1.5 mm thin steel plates, reducing thermal resistance and shortening cooling times [37,38,39]. This modeling framework not only enables accurate prediction of temperature distributions and cooling rates but also provides a robust platform for evaluating different cooling strategies, ranging from traditional straight-drilled channels to conformal cooling designs, thereby supporting optimization of cycle times, minimizing residual stresses, reducing warpage, and improving product quality [40,41].

### 2.5. Experimental Measurement Setup and Data Acquisition

#### 2.5.1. Temperature Measurement Setup

In this study, the mold surface temperature was measured prior to the injection of the polymer melt. This approach was adopted to completely isolate the influence of the heating-cooling system and to eliminate perturbations that could arise from the polymer filling process and the heat exchange at the mold-polymer interface. This methodology ensures that the acquired data accurately reflect the intrinsic thermal response characteristics of the mold surface under controlled heating conditions, independent of the complex dynamics of polymer flow.

**Figure 7:**
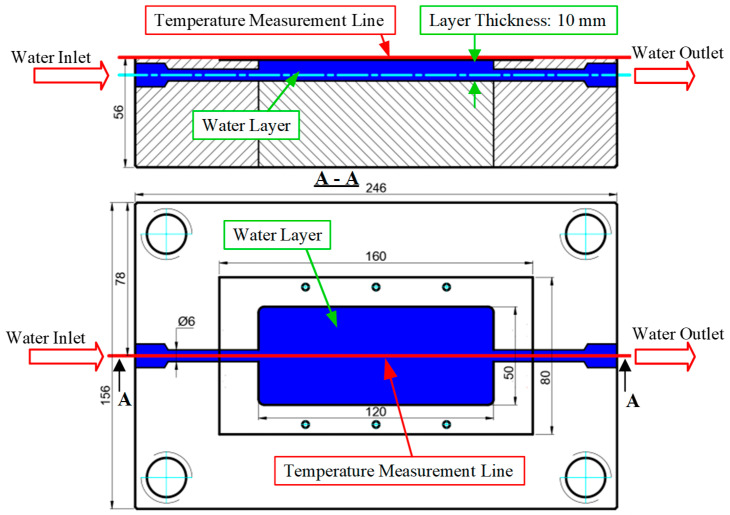
Schematic of the cooling water layer layout and the temperature measurement line in the injection mold.

The strategic placement of temperature sensors within the cooling layer (Figure 7) is pivotal for both process monitoring and design optimization, providing critical data for analyzing heat transfer efficiency. The cooling channels are arranged in parallel and coaxially along the cavity length to foster stable coolant circulation, mitigate the formation of flow dead zones, and promote a uniform temperature distribution. A nominal thickness of 10 mm was maintained for the cooling layer. This dimension is a critical parameter that governs the system’s overall thermal resistance, as it dictates both the heat conduction path from the molding surface to the coolant and the mold’s mechanical load-bearing capacity. This value represents a deliberate trade-off between heat dissipation efficiency, which favors a thin layer, and structural integrity, which necessitates a thick layer. This arrangement minimizes lateral thermal gradients and positions the cooling channels proximate to regions of high heat generation, thereby reducing part warpage and non-uniform shrinkage.

The temperature data acquisition points are arranged linearly at the central position on the mold surface, oriented along the cooling layer, to collect reliable data on the distribution of and variation in the thermal field during each molding cycle. The selection of these measurement locations is critical as it allows for the close monitoring of temperature changes in regions subjected to high thermal loads, thereby facilitating the comparison and calibration of simulation results with experimental data. Furthermore, the data collected from these points provide a basis for assessing the mold’s temperature uniformity, optimizing the cooling channel design and operating conditions, and mitigating the risk of warpage and non-uniform shrinkage in the final product. Thus, the schematic in the figure not only provides a clear illustration of the cooling layer layout but also plays a crucial role in analyzing the temporal temperature distribution and calibrating the thermal model of the injection mold.

#### 2.5.2. Experimental Equipment

**Figure 8:**
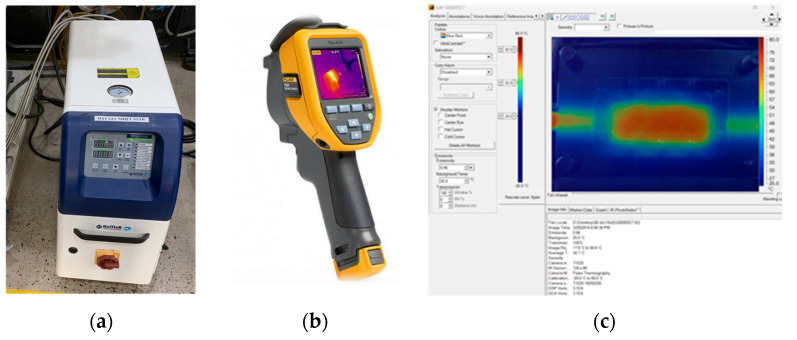
Experimental equipment used in the mold heating and thermal measurement process. (**a**) Mold Heating Unit (Haitian) [45]. (**b**) Fluke Ti20 Infrared Thermal Camera [46]. (**c**) SmartView 4.4 software used for thermal image analysis and post-processing [47].

In this study, the mold surface temperature measurement system was designed based on the integration of three specialized instruments to ensure both accuracy and reliability of the collected data. First, a Haitian mold heater was employed to maintain and control the mold temperature throughout the experiments, providing stable thermal boundary conditions for accurate measurement [42]. Second, a Fluke Ti20 infrared (IR) thermal camera was utilized to capture the temperature distribution on the mold surface at predefined sampling locations. This device enables non-contact measurements with high sensitivity and real-time thermal imaging capability, thereby supporting a precise assessment of heat transfer behavior within the mold [43]. Finally, SmartView 4.4 software was used to process, calibrate, and analyze the thermal data acquired from the IR camera. Through this software, detailed thermal maps were generated, enabling a comprehensive comparison between experimental measurements and numerical simulation results [44]. The integration of these three components establishes an effective thermal measurement framework, ensuring that the experimental data accurately reflect the thermal state of the mold under injection molding conditions (Figure 8).

#### 2.5.3. Injection Molding Experimental Setup

**Figure 9:**
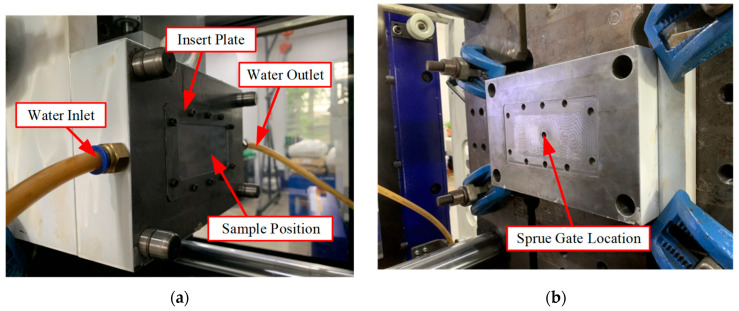
Photographs of the experimental setup and structural components of the mold insert system. (**a**) Core mold. (**b**) Cavity mold.

For the molding trials, the validated insert mold system was mounted onto a Haitian MA1200 III injection molding machine (Figure 9). The mold was connected to the water supply and return circuits for dynamic thermal cycling during the molding process. As shown in Figure 9a, the core side features an integrated cooling system with an inlet and outlet to circulate the heat transfer medium, thereby maintaining stable thermal conditions. The cavity side (Figure 9b) is equipped with a central sprue gate that directs the molten polypropylene (PP) into the runner system, ensuring uniform distribution. The operational sequence leveraged this configuration as follows: initially, hot water circulation rapidly elevated the mold temperature to the desired setpoint, which enhanced melt flowability and minimized internal stresses during filling. Subsequently, cold water was circulated through the same channels to rapidly reduce the mold temperature, thereby promoting structural stability during solidification. This integrated configuration not only shortens the injection molding cycle time but also replicates industrial operating conditions with high fidelity. The system’s high repeatability and reliability yielded consistent samples, enabling an accurate performance evaluation.

## 3. Results and Discussion

### 3.1. Mold Heating Analysis at 70 °C

**Figure 10:**
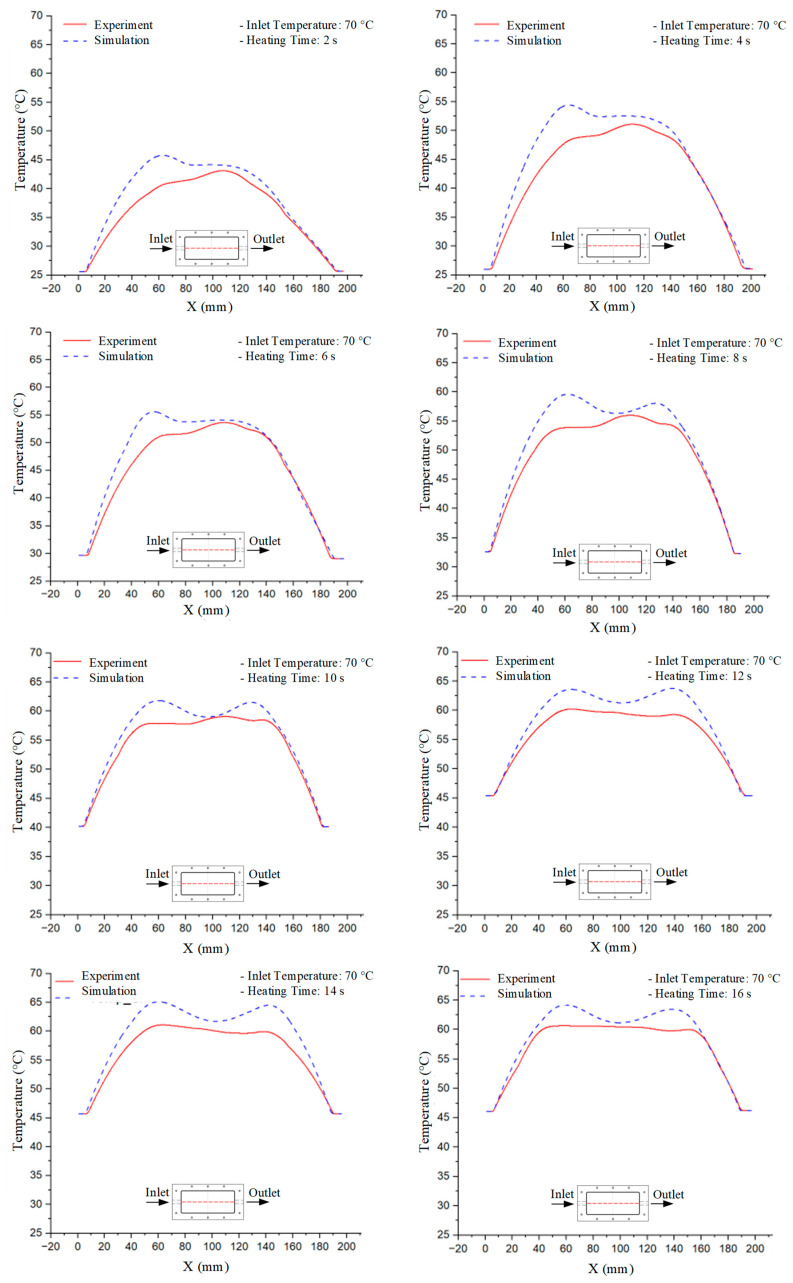

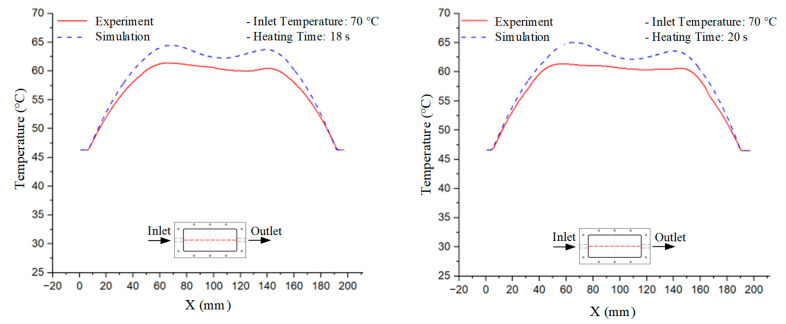
Experimental and simulation temperature distributions in the cavity at an inlet temperature of 70 °C under different heating times (2–20 s).

**Figure 11:**
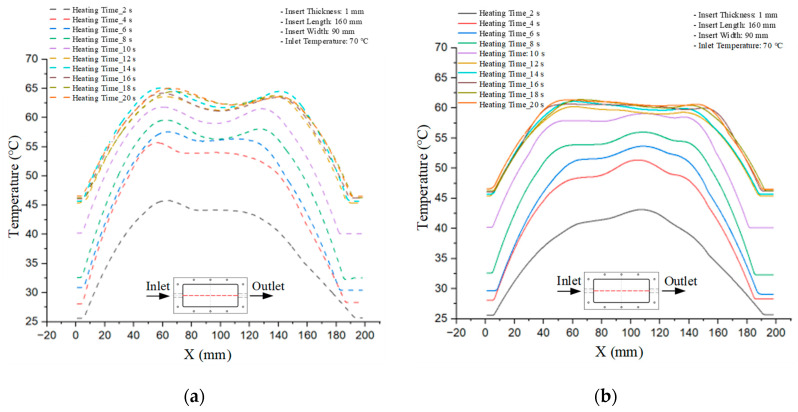
Consolidated temperature distributions in the cavity wall at 70 °C obtained by combining the results in Figure 10: (**a**) simulation data, (**b**) experimental data.

Mold Heating Analysis at 70 °C results presented in Table 1, Figure 10 and Figure 11.

### 3.2. Mold Heating Analysis at 80 °C

**Figure 12:**
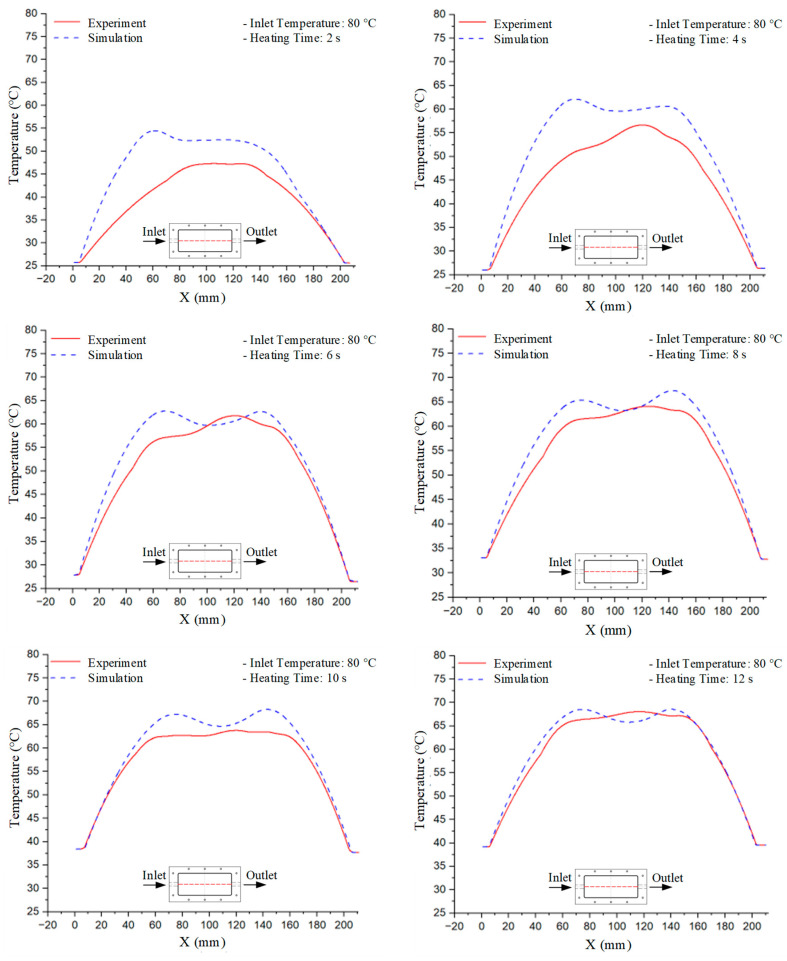

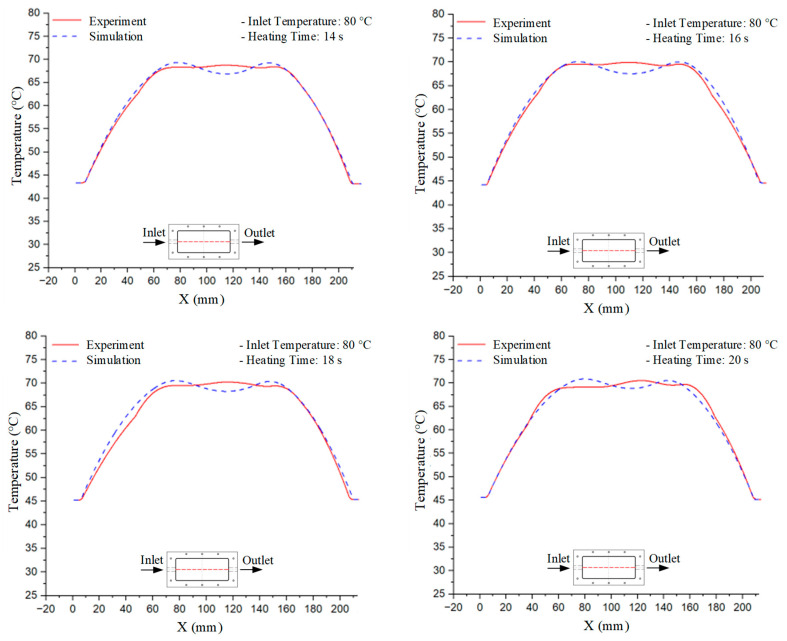
Experimental and simulation temperature distributions in the cavity at an inlet temperature of 80 °C under different heating times (2–20 s).

**Figure 13:**
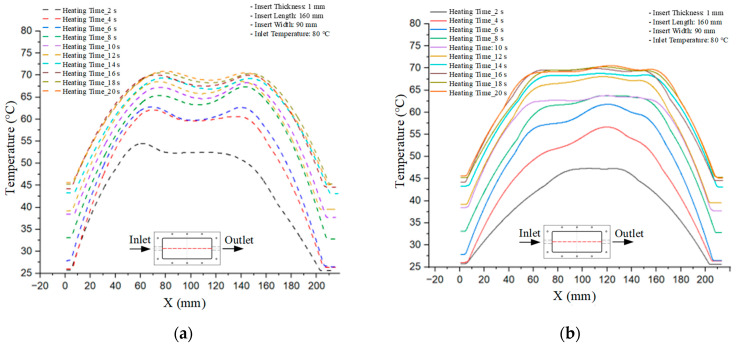
Consolidated temperature distributions in the cavity wall at 80 °C obtained by combining the results in Figure 12: (**a**) simulation data, (**b**) experimental data.

Mold Heating Analysis at 80 °C results presented in Table 2, Figure 12 and Figure 13.

### 3.3. Mold Heating Analysis at 90 °C

**Figure 14:**
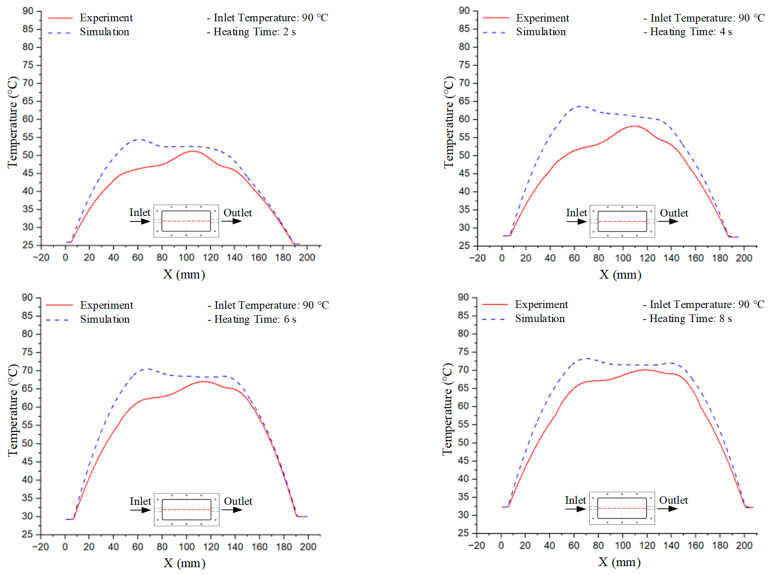

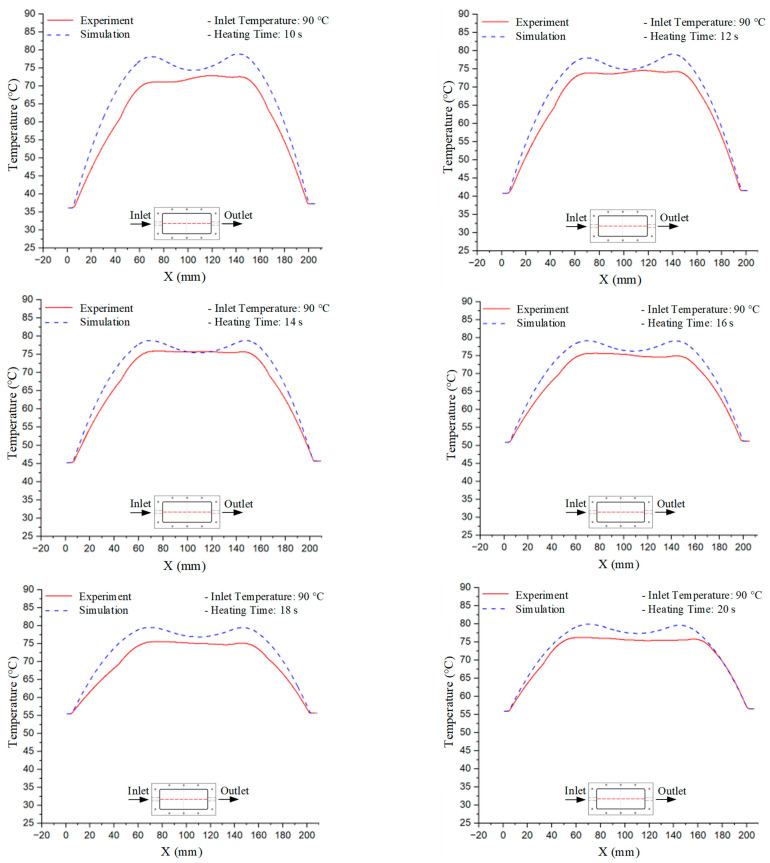
Experimental and simulation temperature distributions in the cavity at an inlet temperature of 90 °C under different heating times (2–20 s).

**Figure 15:**
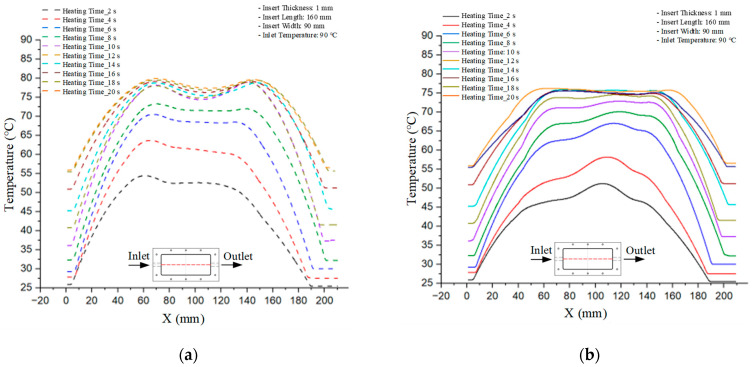
Consolidated temperature distributions in the cavity wall at 90 °C obtained by combining the results in Figure 14: (**a**) simulation data, (**b**) experimental data.

Mold Heating Analysis at 80 °C results presented in Table 3, Figure 14 and Figure 15.

### 3.4. Comparative Analysis Across Different Heating Conditions

The investigation at three temperature setpoints (70 °C, 80 °C, and 90 °C), with the results presented in Table 1, Table 2 and Table 3, reveals a consistent three-stage trend in the temporal evolution of the mold temperature. In the initial stage (2–6 s), the temperature curve exhibits a rapid increase with a steep slope, reflecting the instantaneous heat absorption at the mold surface upon exposure to the heating medium. The discrepancy between simulation and experimental results is typically highest during this phase, influenced by initial thermal gradients and boundary condition assumptions that may not fully capture the actual heat transfer process. In the intermediate stage (8–12 s), the rate of temperature increase gradually slows as it approaches a quasi-equilibrium state. Here, the deviation between simulation and experiment narrows considerably as heat conduction within the mold block becomes more stable, minimizing the impact of local errors. Finally, in the saturation stage (14–20 s), the mold temperature converges toward a steady state, with a near-zero rate of change and a minimal discrepancy between the two datasets. This three-stage trend is replicated consistently across all three investigated setpoints, demonstrating the stability and reliable predictive capability of the simulation in describing the heat transfer kinetics within the injection mold. Thus, although the absolute temperature setpoints differ, the temporal profile of temperature variation maintains a similar dynamic structure, providing a critical basis for optimizing and comparing different operating conditions in both research and industrial applications.

For a quantitative analysis of the thermal distribution within the cooling layer system—along the path from the water inlet, through the heated layer, to the water outlet—a comparison between simulation and experimental results at discrete time points from 2 to 20 s was conducted for the three setpoints (Figure 10, Figure 11, Figure 12, Figure 13, Figure 14 and Figure 15). The results show a distinct evolution of the thermal field over time, as well as a notable discrepancy between the simulation and experimental data. In the initial stage (2–6 s), the temperature rise exhibits a clear trend where the simulation results consistently over-predict the experimental values. Specifically, the peak average temperatures in the central region reached 45.6–55.6 °C, 50.4–64.8 °C, and 53.4–70.4 °C in the simulation for the 70 °C, 80 °C, and 90 °C setpoints, respectively. In contrast, the experimental results recorded corresponding values of 43.1–53.6 °C, 47.3–61.8 °C, and 51.2–68.0 °C.

This discrepancy suggests that the numerical model tends to predict a faster heating rate during the onset, likely due to an incomplete representation of heat loss to the ambient environment and the thermal inertia of the sensors during measurement. The maximum observed errors during this period were 3.3 °C (6.46%), 3.1 °C (6.55%), and 4.5 °C (7.61%) for the three setpoints. Although the absolute and relative errors in this stage exceed 6%—higher than in subsequent stages due to the low-temperature range, short duration, delayed thermal response, and the thermal resistance of the insert impeding heat transfer—they remain within the acceptable limits for experimental heat transfer studies in injection molding. This outcome indicates that, despite the quantitative differences, the simulation model accurately captures the dynamic trend of the initial heating phase. This provides a crucial basis for calibrating boundary parameters and enhancing the reliability of thermal distribution predictions under real-world operating conditions.

In the intermediate stage (8–12 s), as heating progresses, both simulation and experimental results show a convergence toward a thermal steady state. The average temperatures obtained from the simulation were approximately 59–63.7 °C, 67.3–68.5 °C, and 73.2–79 °C for the 70 °C, 80 °C, and 90 °C setpoints, respectively. In contrast, the experimental results reached only 56–60.2 °C, 64.7–68.0 °C, and 72.1–77.5 °C. This deviation reflects the error between the two methods, with maximum values recorded in this stage of 3.5 °C (5.81%), 2.6 °C (4.02%), and 1.5 °C (1.94%). Notably, the error remains relatively high at the lower setpoint (70 °C), likely due to the significant influence of ambient heat loss and thermal lag in the measurement system. Conversely, at higher setpoints (80 °C and 90 °C), the discrepancy between simulation and experiment diminishes significantly, indicating better convergence as thermal stability is established. This trend indicates that the simulation accurately predicts the heat transfer kinetics, with its reliability improving at higher setpoints where exogenous effects are minimized. This is significant for optimizing operating parameters, as it allows for more accurate predictions of heat transfer efficiency and heating times in production environments.

In the final stage (14–20 s), the difference between simulation and experimental results is substantially reduced, indicating the system has nearly reached a thermal steady state. The peak average temperatures in the central region in the simulation reached 63.7 °C, 68.5 °C, and 79.0 °C for the three respective setpoints. The corresponding experimental values were 60.2 °C, 68.0 °C, and 77.5 °C. The maximum observed errors in this stage were 4.0 °C (6.55%), 0.6 °C (0.87%), and 1.0 °C (1.27%). These results demonstrate that, despite minor quantitative differences, the numerical simulation almost completely and accurately describes the heat transfer behavior in the mold during the steady-state phase. The low errors (less than 2%) at 80 °C and 90 °C confirm a high correlation between simulation and experiment as the system reaches equilibrium, while the higher error at 70 °C can be attributed to the more pronounced impact of external heat loss mechanisms under low-temperature conditions. Overall, the analysis of this final stage demonstrates that the numerical simulation not only captures the correct thermal kinetics but also accurately reflects the system’s thermal stability, providing a reliable basis for its application in the design optimization and operation of injection molds in industrial practice.

**Figure 16:**
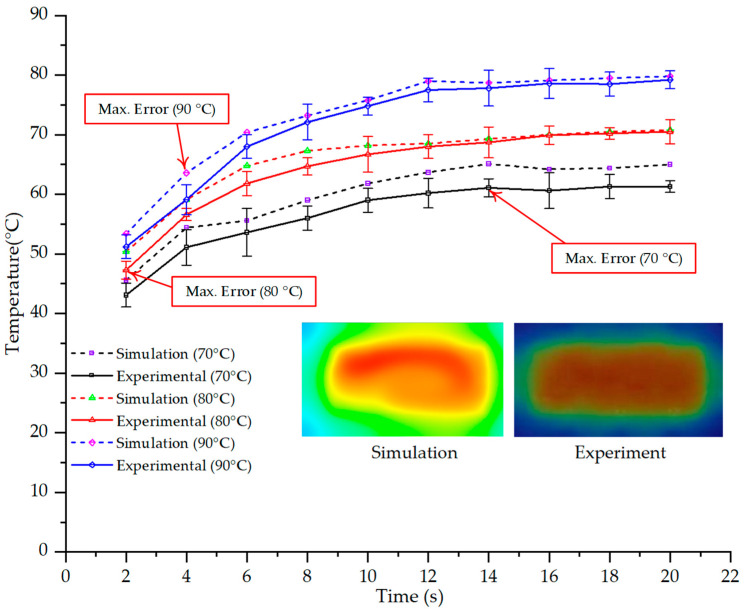
Maximum temperature over time under different heating conditions: simulation vs. experimental results.

The analysis of the three investigated temperature setpoints (70 °C, 80 °C, and 90 °C), detailed in Table 4 and Figure 16, reveals a consistent three-stage heat transfer trend: initial, intermediate, and saturation. This reflects the characteristic thermal kinetics of the injection molding process, where the initial thermal state is highly dynamic but gradually converges toward equilibrium over time. In the initial stage (2–6 s), the rate of temperature increase in the simulation consistently exceeds that of the experiment. This phenomenon can be attributed to the idealized boundary conditions assumed in the numerical model, whereas in reality, heat loss mechanisms exist through convection, radiation, and mechanical conduction at the mold-machine interface. Concurrently, the thermal inertia of the sensors causes a lag in the experimental data. Consequently, the maximum error in this stage reaches 6–7%, significantly higher than in subsequent stages [48]. Moving to the intermediate stage (8–12 s), the thermal system approaches a quasi-equilibrium state. The simulated and experimental temperature values gradually converge, with the error decreasing to approximately 2–5%. Notably, at higher temperatures (80–90 °C), this deviation diminishes more markedly, demonstrating that the influence of exogenous heat loss and sensor lag becomes less significant as the thermal gradient decreases [49]. This trend is consistent with previously published works, which typically report the largest discrepancies between simulation and experiment in the initial phase, whereas in the steady state, simulations accurately reflect measured data [50]. In the saturation stage (14–20 s), the mold temperature reaches a steady state with a near-zero rate of change. The absolute error becomes very small, ranging from 0.6 to 4.0 °C, which corresponds to a relative error below 2% at 80 °C and 90 °C. This indicates that the simulation model is highly reliable in describing the steady-state thermal condition. This convergence is critically important for industrial applications, as the steady-state phase directly governs final product quality (e.g., shrinkage, warpage, surface gloss) and the molding cycle time [51].

Another noteworthy finding is the correlation between mold temperature and injection pressure. The results show that as mold temperature increases, melt viscosity decreases, thereby reducing the injection pressure required to achieve an equivalent flow length. Conversely, if the mold temperature drops rapidly due to thin walls and localized cooling, the required injection pressure must increase sharply to compensate for the reduced fillability, which can easily induce residual stresses and product deformation. This underscores the pivotal role of the Cooling Layer in maintaining surface temperature uniformity, which helps to control injection pressure at a reasonable level and simultaneously reduces the risk of defects [5]. In summary, the research indicates that the numerical simulation model can reliably predict the heat transfer evolution in a mold equipped with a Cooling Layer, particularly during the steady-state phase and at high temperatures. Although quantitative errors exist in the initial stage, they fall within the acceptable limits reported in previous experimental studies [52,53,54]. This reinforces the potential of a combined simulation and experimental approach as an effective tool for optimizing the design and operation of industrial injection molds.

### 3.5. Experimental Injection Molding

A primary challenge in validating numerical simulations of injection molding is the discrepancy that often arises between predicted and experimental results. These deviations stem from the complex, coupled phenomena inherent to the process—including heat transfer, turbulent flow, and nonlinear material properties—and the idealized boundary conditions typically employed in simulation software. To mitigate this limitation, this study employs an experimental approach using two distinct benchmark molds: a “Partial tooth” sample and a “Hook-shaped leaf” sample, which differ in runner count and cavity geometry. This dual-configuration method facilitates cross-comparison and allows for an assessment of the simulation’s sensitivity to variations in geometry and flow distribution. From a theoretical perspective, while mold temperature distribution directly influences product quality, numerical tools often assume a uniform heat transfer coefficient. In reality, the coolant flow is frequently non-uniform, and additional factors like radiative and convective losses contribute to the observed discrepancies.

Through parallel experiments with these two molds, this study not only elucidates the mechanism of symmetric heat spreading toward the cavity sidewalls but also establishes a quantitative basis for assessing modeling errors. This integrated methodology ultimately strengthens the correlation between simulation and experiment, providing a reliable framework for optimizing cooling layer design, boundary condition modeling, and process control in injection molding.

For the injection molding experiments, the selected material was a polypropylene (PP) homopolymer, grade Advanced-PP 1100 N, sourced from Advanced Petrochemical Company (Al Jubail, Saudi Arabia). As specified in the manufacturer’s technical datasheets (Table 5 and Table 6), the properties of this material guarantee stability during the molding process and affirm its suitability for the investigation of mold temperature effects.

#### 3.5.1. Case 1: Partial Tooth Sample

**Figure 17:**
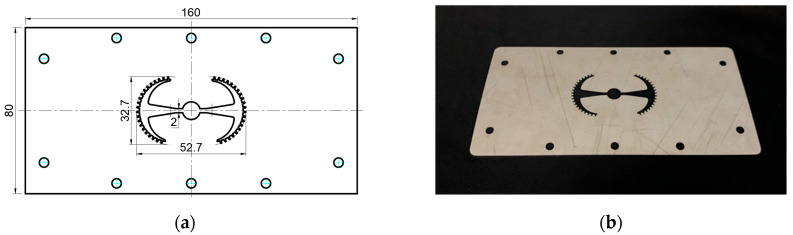
(**a**) Design drawing of the insert plate with a partial tooth sample. (**b**) Fabricated insert plate.

**Figure 18:**
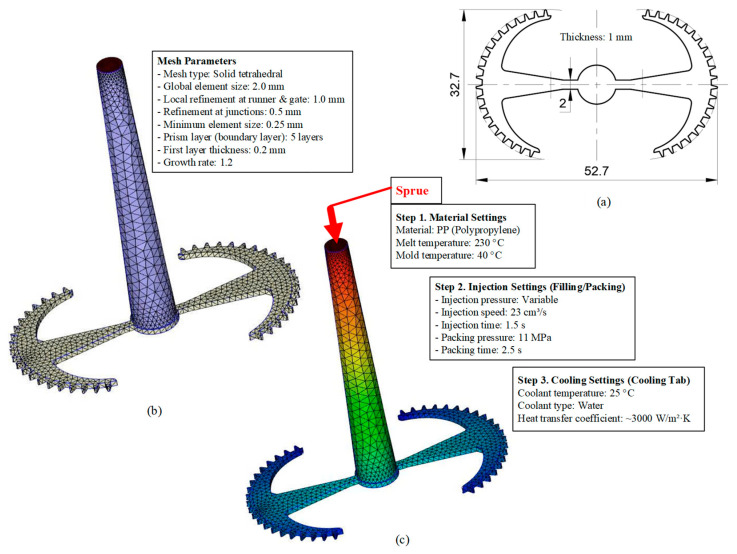
Partial tooth mold: (**a**) Technical drawing of the molded part; (**b**) Finite element mesh of the sprue and part; (**c**) Simulation setup showing runner and sprue geometry, material properties, injection settings, cooling settings, and mesh parameters.

**Figure 19:**
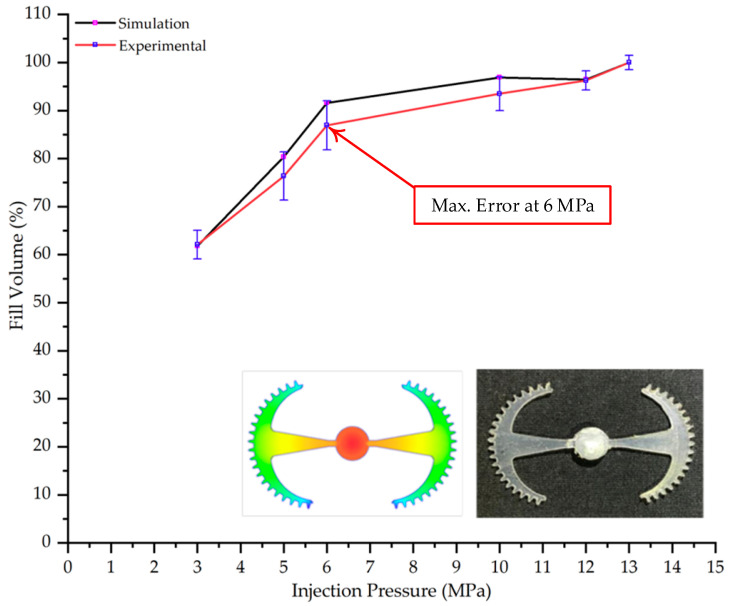
Comparison of simulation and experimental fill volume (%) at different injection pressures for PP.

The results obtained from Figure 17, Figure 18 and Figure 19 and Table 7 and Table 8 reveal significant differences in the filling behavior of the “Partial tooth” mold under varying injection pressures ranging from 3 MPa to 13 MPa. By integrating both numerical simulations and experimental measurements, it is evident that increasing the injection pressure considerably reduces the occurrence of short shots and substantially improves overall product quality. At the initial low-pressure stage of 3 MPa, the simulation predicted a filling volume of 61.80%, while the experimental result recorded 62.09%, corresponding to a deviation of only 0.46%; this minor difference arises because the kinetic energy of the molten polymer is insufficient to overcome the flow resistance along the runner, causing a slower flow front advancement compared to the idealized conditions in the simulation. When the pressure increased to 5 MPa, the filling volume rose sharply to 80.38% in the simulation and 76.36% experimentally (5.27% deviation), as a noticeable thermal gradient formed along the runner, causing localized viscosity reduction and introducing a velocity imbalance between the branches. In the medium-pressure range of 6–10 MPa, filling efficiency improved significantly, with the deviation reduced to 3.64% at 10 MPa. This improvement reflects the role of heat transfer and pressure balancing at branching points, which reduces the influence of vortices and results in a more stable, symmetric filling pattern as reported in prior studies. At the high-pressure stage of 12–13 MPa, both the simulation and experiment achieved nearly 100% filling with deviations below 1%, indicating that the melt flow transitioned into a quasi-saturated regime where the impact of nonlinear viscosity is significantly reduced. However, it is important to note that such elevated pressures can induce higher residual stresses in molded products, emphasizing the necessity of a balanced process design to optimize filling completeness alongside part quality and stress management.

A comparative analysis provides deeper insight into these dynamics, revealing a distinct trend of heat propagation from the gate toward the cavity sidewalls that was particularly pronounced in the experimental results (Table 7 and Table 8). This observation, further illustrated by the thermal distribution in Figure 18, highlights the significant influence of real-world thermal contact conduction and convective heat transfer, effects that are often idealized in simulations and lead to discrepancies. As shown in Figure 19, while the fill volume ratio exhibits a near-linear increase with injection pressure, the experimental slope is consistently 6–8% lower than simulation predictions in the intermediate range, demonstrating that localized heat loss and flow balancing remain the dominant controlling factors in real conditions. Overall, the results suggest that improving the filling uniformity of the “Partial tooth” mold requires a synergistic approach integrating three key strategies: (i) optimizing runner geometry to minimize flow resistance, (ii) adjusting mold temperature profiles to reduce local thermal gradients, and (iii) calibrating numerical models with experimental datasets to enhance predictive accuracy. These findings are consistent with previous studies on multi-cavity runner design, which similarly reported improvements in filling balance and thermal uniformity through integrated experimental and simulation-based approaches.

#### 3.5.2. Case 2: Hook-Shaped Leaf Sample

**Figure 20:**
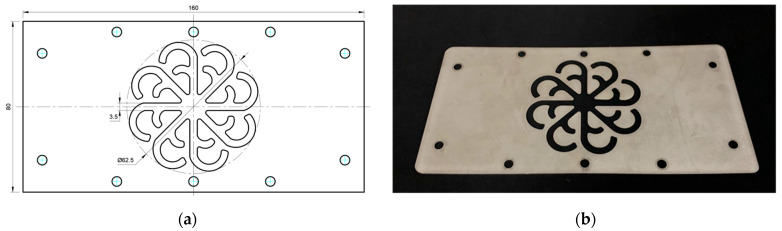
(**a**) Design drawing of the insert plate with a hook-shaped leaf sample. (**b**) Fabricated insert plate.

**Figure 21:**
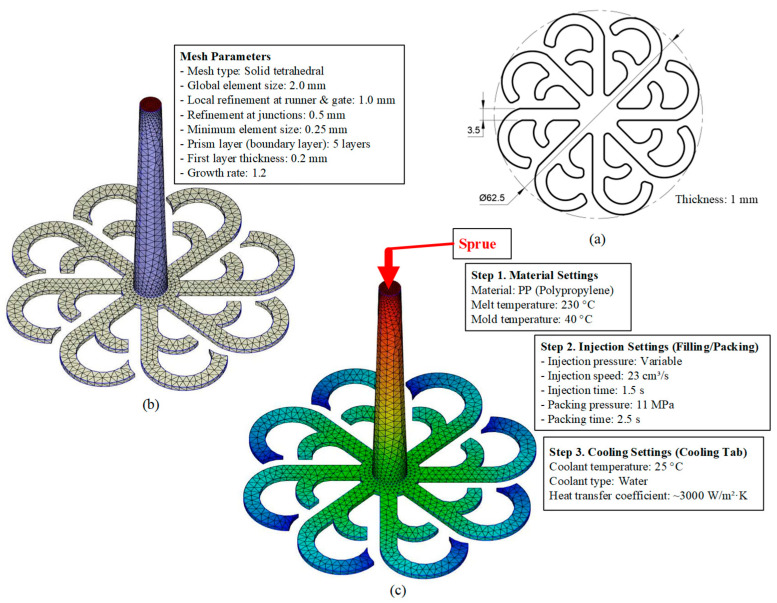
Hook-shaped leaf mold: (**a**) Technical drawing of the molded part; (**b**) Finite element mesh of the sprue, runners, and part; (**c**) Simulation setup showing runner and sprue geometry, material properties, injection settings, cooling settings, and mesh parameters.

**Figure 22:**
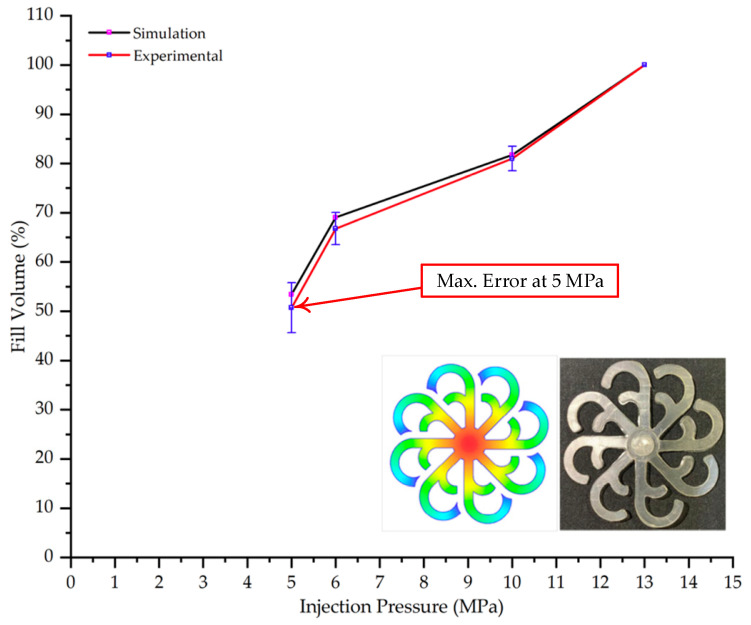
Comparison of fill volume (%) between simulation and experimental results at different injection pressures for PP material.

The results presented in Figure 20, Figure 21 and Figure 22 and Table 9 and Table 10 illustrate the detailed characteristics of the filling behavior for the hook-shaped leaf mold under injection pressures ranging from 5 MPa to 13 MPa. The integration of numerical simulation and experimental measurements reveals that increasing the injection pressure significantly influences the filling volume, thermal distribution, and flow-front propagation dynamics. At the low-pressure stage of 5 MPa, the predicted filling volume from the simulation reached 53.42%, while the experimental measurement was 50.71%, resulting in a deviation of approximately 5.34%. This discrepancy arises from the assumption of idealized boundary conditions and a uniform heat transfer coefficient in the simulation, whereas the experiment accounts for localized heat losses that increase melt viscosity and hinder flow progression, leading to short-shot defects as confirmed in Table 9. At the intermediate pressure stage, the filling volume increased significantly; at 6 MPa the error was reduced to 3.40%, and at 10 MPa, the simulation predicted 81.73% filling compared to 81.00% in the experiment, yielding a minimal deviation of 0.9%. This improvement indicates that pressure equilibrium within the runner network had been established, enabling stable and uniform melt flow, which is consistent with prior studies on overcoming runner junction resistance. Supporting evidence from Table 9 demonstrates improved synchronization of cavity filling, while Figure 22 highlights the near-linear relationship between filling volume and injection pressure, attributed to the reduction in melt viscosity as cavity wall temperatures rise. Finally, at the high-pressure stage of 13 MPa, both the simulation and experimental results achieved 100% filling with negligible deviation. However, as reported in recent studies, exceeding the optimal injection pressure may lead to increased residual stresses and warpage, necessitating careful optimization to balance filling completeness, dimensional accuracy, and structural integrity.

The results from Figure 21 reveal a significant difference in thermal distribution during the filling process, which helps explain the performance deviations. In the simulation, higher temperatures are concentrated in the central region of the runner, whereas the experimental data demonstrate a more uniform heat dissipation toward the sidewalls due to real-world mold-contact heat conduction and forced convection—phenomena that are idealized in the simulation model. This thermal discrepancy is a primary factor contributing to deviations in filling volume under low to intermediate injection pressures. Overall, the findings suggest that achieving high-accuracy thermal and flow simulations for the hook-shaped leaf mold requires three critical improvements: (i) calibrating the heat transfer coefficient based on experimental measurements to better reflect real thermal boundary conditions; (ii) optimizing runner design to achieve a more balanced pressure distribution across all cavities; and (iii) integrating nonlinear heat transfer models into the simulation to accurately capture melt viscosity reduction mechanisms. These refinements are essential to enhance the predictive reliability of the simulation, minimize discrepancies with experimental results, and improve mold design optimization for multi-cavity injection molding systems.

## 4. Conclusions

This study quantitatively elucidates that integrating a CL with a thin-walled mold structure mitigates thermal resistance, enhances thermal homogeneity at the mold surface, and improves filling stability during PP injection molding. The numerical model was calibrated via infrared thermography at three inlet water temperatures (70 °C, 80 °C, and 90 °C). The validation revealed that the deviation between the simulation and experiment diminishes over time, converging at steady state. At 70 °C, the maximum deviation at 20 s was approximately 6.04% (65.0 °C vs. 61.3 °C), whereas at 80 °C and 90 °C, the deviations were substantially lower at 0.43% (70.8 °C vs. 70.5 °C) and 0.76% (79.8 °C vs. 79.2 °C), respectively. The principal discrepancies were confined to the initial transient phase (2–12 s). During this period, the simulation predicted a faster temperature rise with a concentrated thermal peak, whereas the experimental data exhibited symmetric thermal diffusion toward the cavity walls, a difference attributable to real-world conduction–convection mechanisms and heat loss to the ambient environment.

From a process perspective, injection molding trials on the “Partial tooth” and “Hook-shaped leaf” configurations confirmed the critical role of uniform thermal fields in governing flow-front dynamics. For the “Partial tooth” mold, the fill volume ratio increased in a near-linear fashion with injection pressure: from approximately 62% at 3 MPa to 93–97% at 10 MPa, where the simulation–experiment deviation was reduced to 3.64% and approached 100% fill at 12–13 MPa (deviation less than 1%). For the “Hook-shaped leaf” mold, a similar trend was observed but required higher pressures to achieve balanced flow in its multi-cavity branches; the deviation decreased from 5.34% at 5 MPa to 0.9% at 10 MPa and approached 0% at 13 MPa. These findings elucidate a strong thermo-fluid coupling effect, where the cooling layer planarizes temperature gradients, reduces localized viscosity, minimizes pressure deficits in distant branches, and ultimately enhances both cavity filling performance and process reproducibility.

Overall, the combined application of a cooling layer and thin-wall mold design significantly enhances prediction reliability (steady-state deviation less than 1% at 80–90 °C) and process efficiency (high filling completeness at medium-to-high pressures). This provides a robust foundation for the design and fabrication of thin-wall injection molds, especially for difficult-to-fill polymer products. Future work should focus on calibrating local heat transfer coefficients under different flow regimes and extending the evaluation to include residual stresses and post-molding mechanical properties to achieve comprehensive process optimization and quality assurance.

From a practical standpoint, these findings can directly inform the structural optimization of Cooling Layers for thin-walled molding applications. Specifically, maintaining a homogeneous temperature field and reducing thermal gradients reduces the injection cycle time while simultaneously elevating product quality by mitigating defects such as warpage, weld lines, and surface imperfections. Moreover, the simulation model demonstrated high fidelity with the empirical results, positioning it as a valuable Computer-Aided Engineering (CAE) tool. This model can be employed to select optimal operating parameters, thereby reducing the economic burden of extensive physical prototyping.

While this study provides valuable insights, its limitations should be acknowledged to contextualize the findings. Notably, the research scope was confined to a planar geometry under a limited set of temperature setpoints and boundary conditions, as the study did not extend to intricate topographies, residual stress analysis, fatigue life (maximum duty cycles), or long-term tool deformation. Furthermore, a simplified physics model was employed that did not fully integrate the effects of diverse mold materials, the nonlinear thermo-mechanical behavior of the polymer, or the complex turbulent flow dynamics within the cooling channels. These areas represent essential avenues for future investigation, and addressing these factors will be crucial to further refine the scientific basis of this work and broaden its practical implementation.

## Figures and Tables

**Table 1:** Simulation and experimental results of mold heating process at 70 °C.

Time (s)	Simulation Results (°C)	Experimental Results (°C)
2	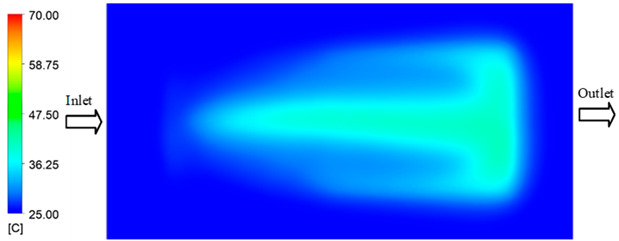	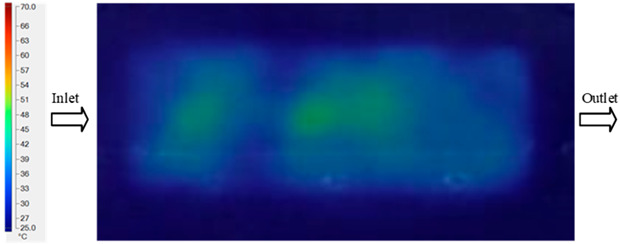
4	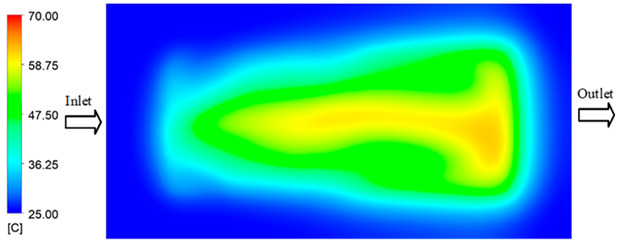	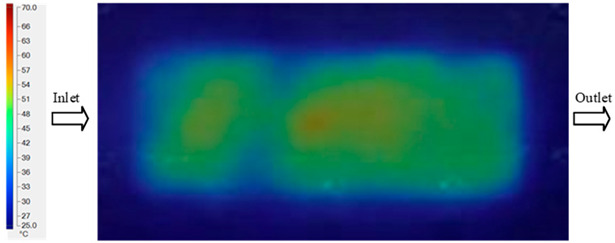
6	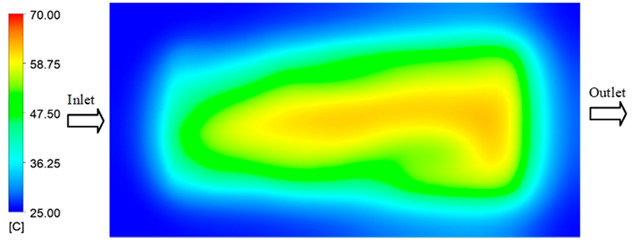	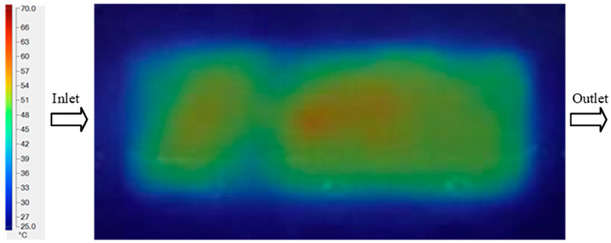
8	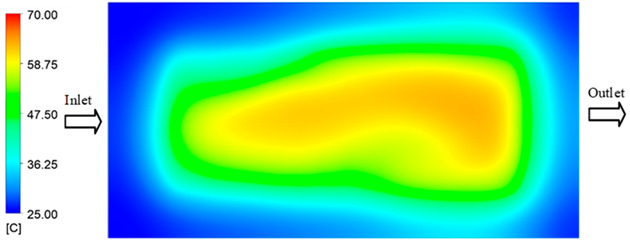	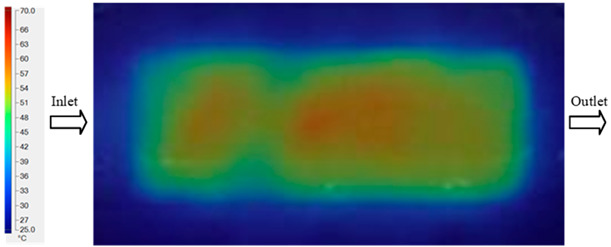
10	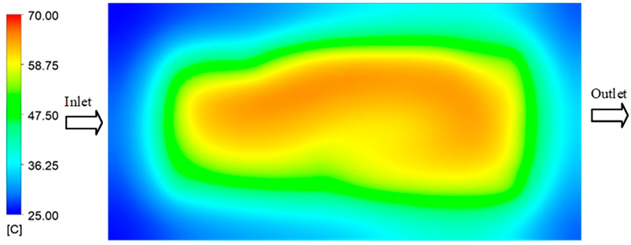	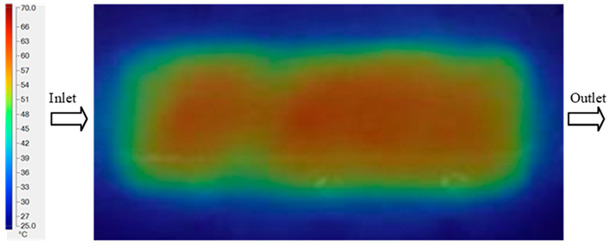
12	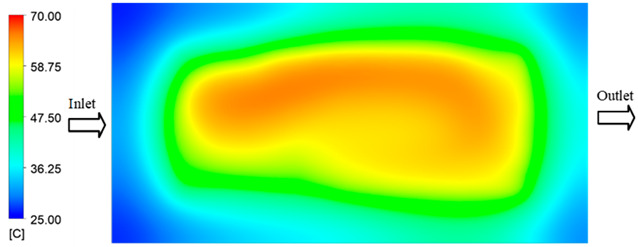	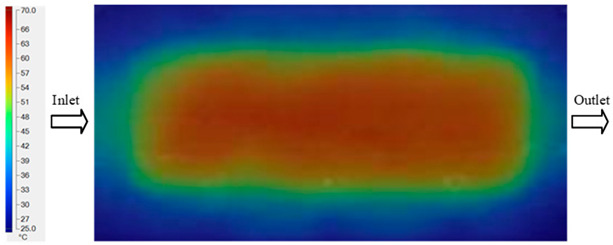
14	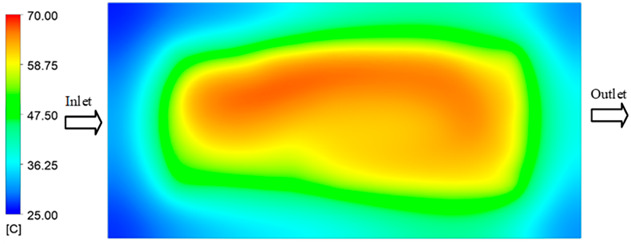	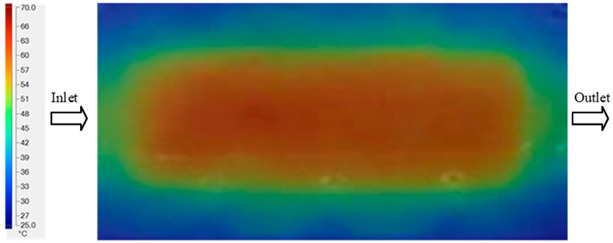
16	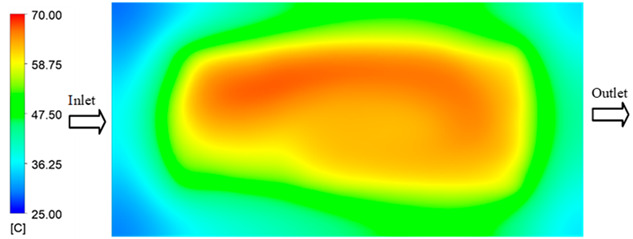	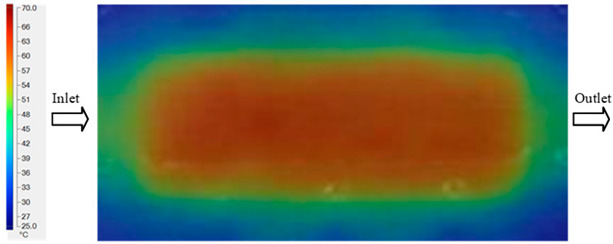
18	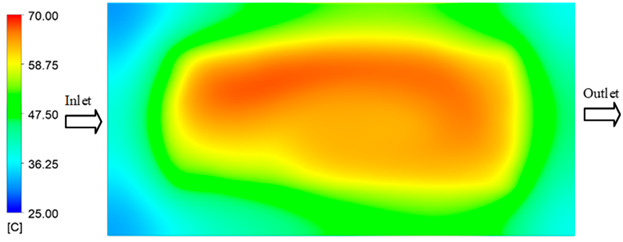	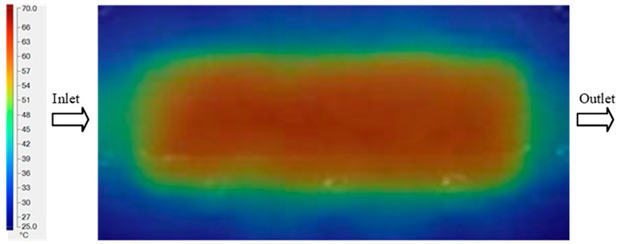
20	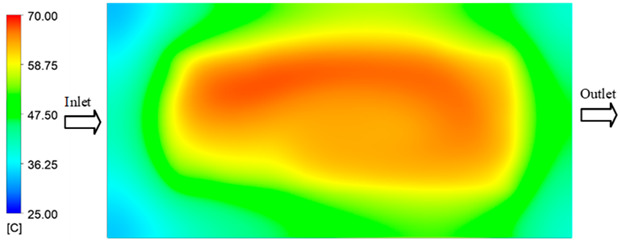	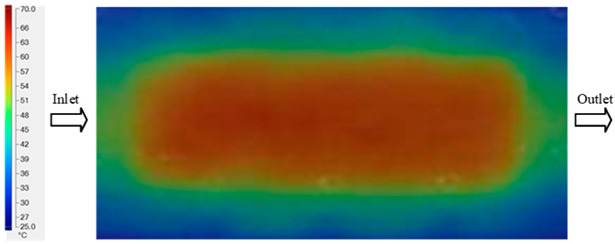

**Table 2:** Simulation and experimental results of mold heating process at 80 °C.

Time (s)	Simulation Results (°C)	Experimental Results (°C)
2	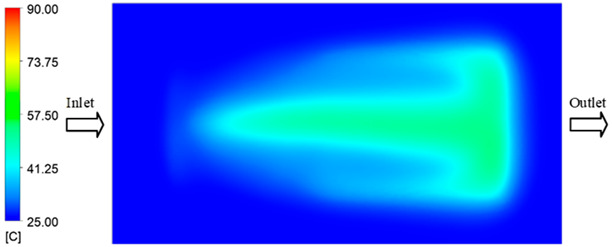	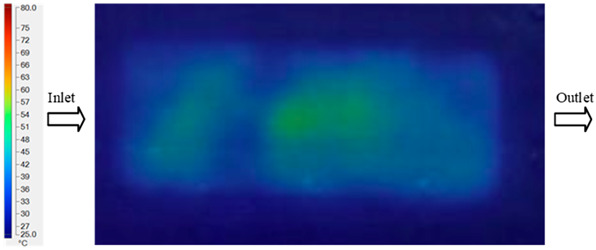
4	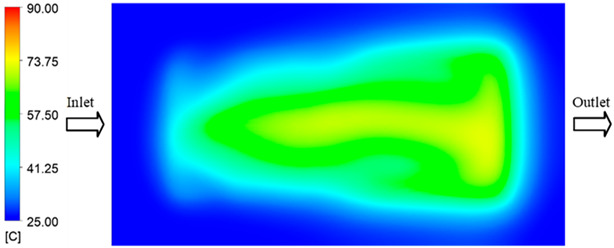	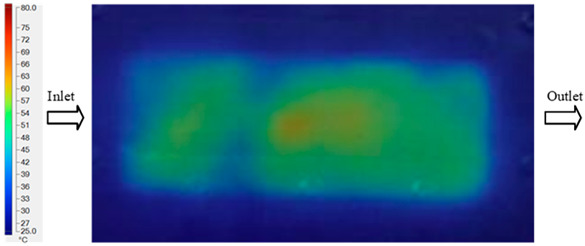
6	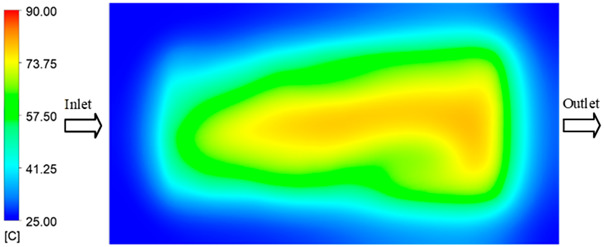	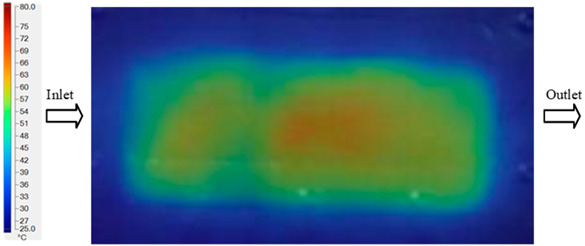
8	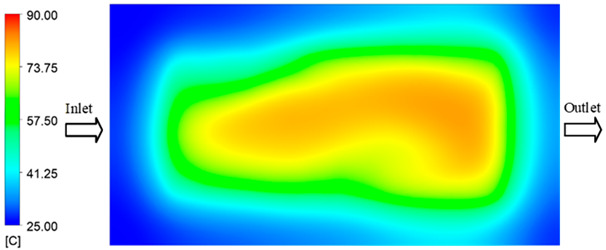	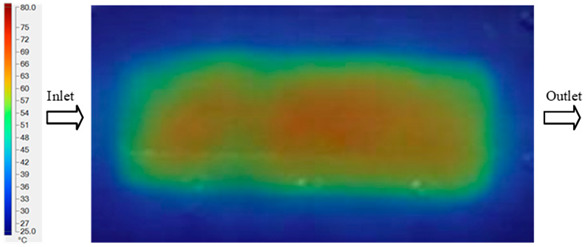
10	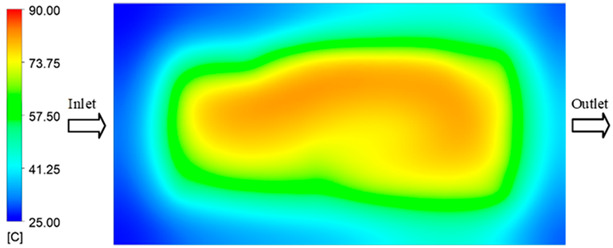	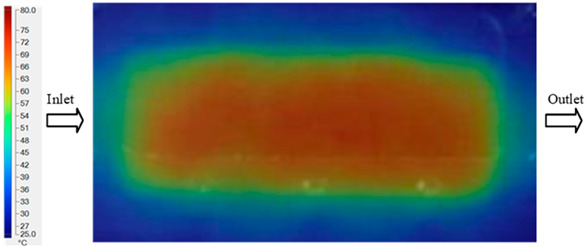
12	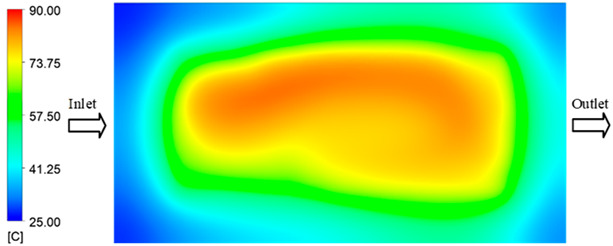	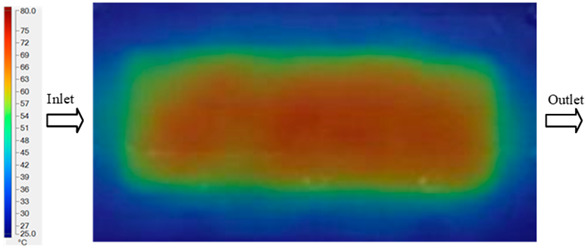
14	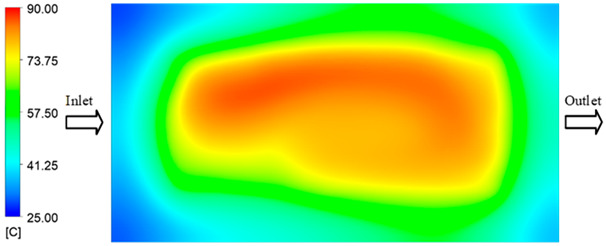	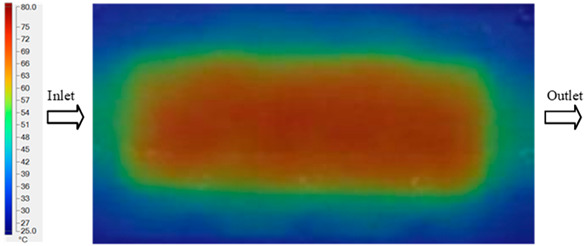
16	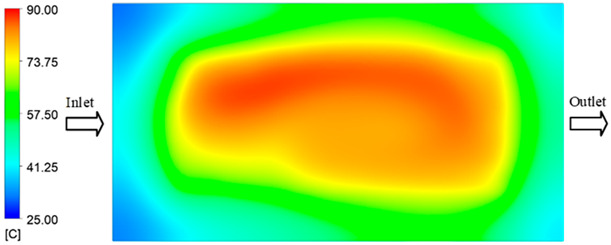	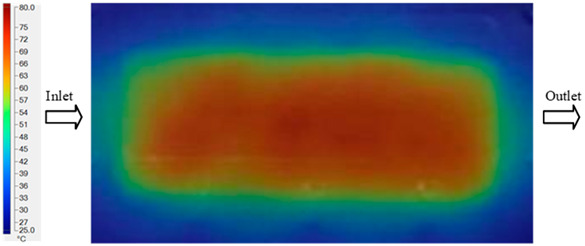
18	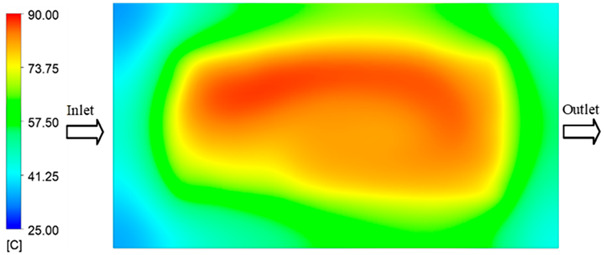	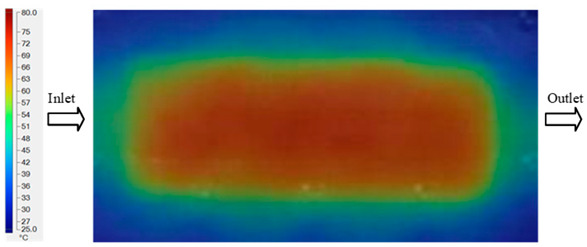
20	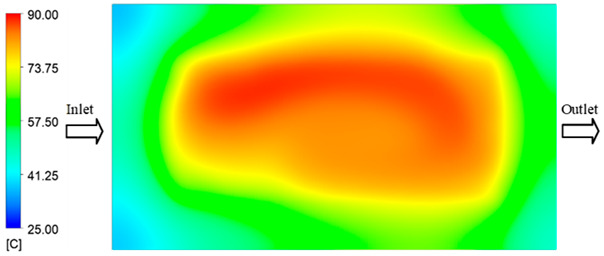	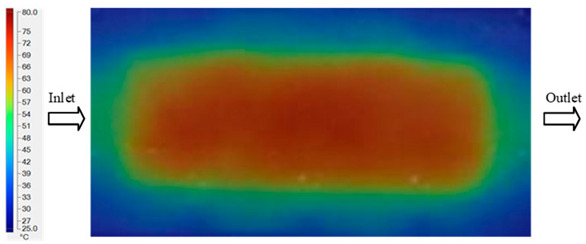

**Table 3:** Simulation and experimental results of mold heating process at 90 °C.

Time (s)	Simulation Results (°C)	Experimental Results (°C)
2	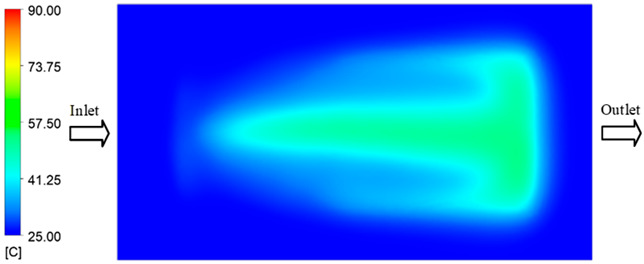	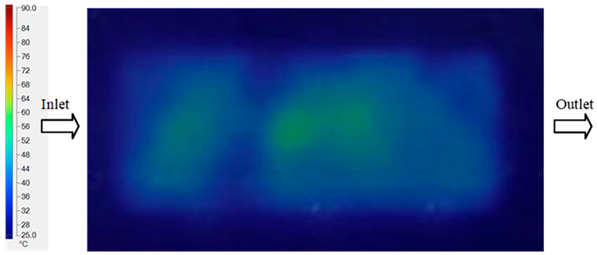
4	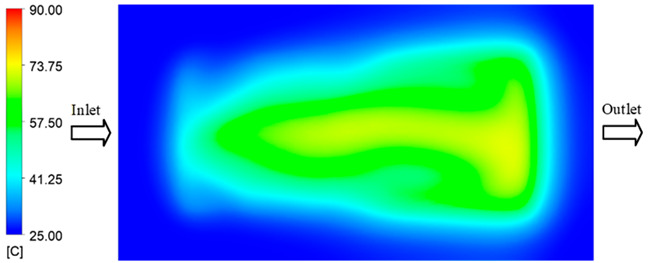	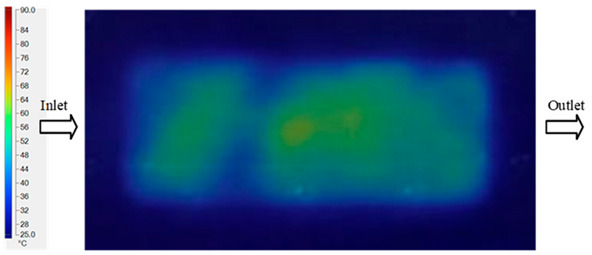
6	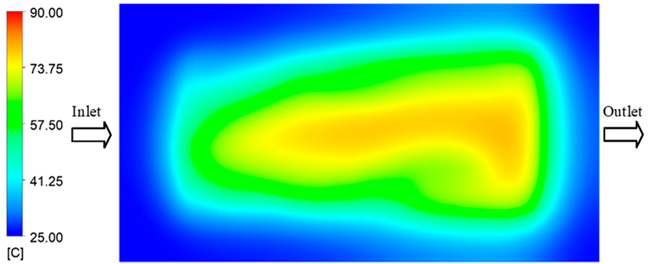	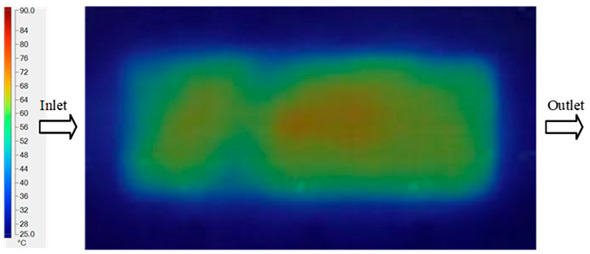
8	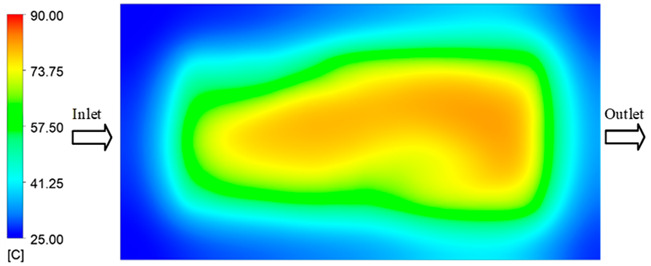	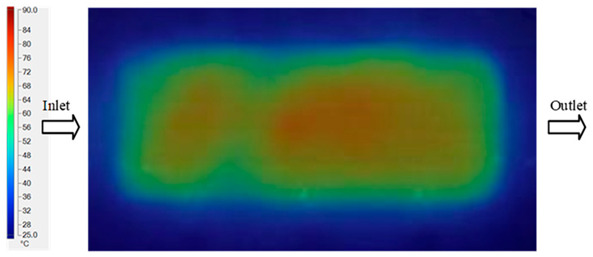
10	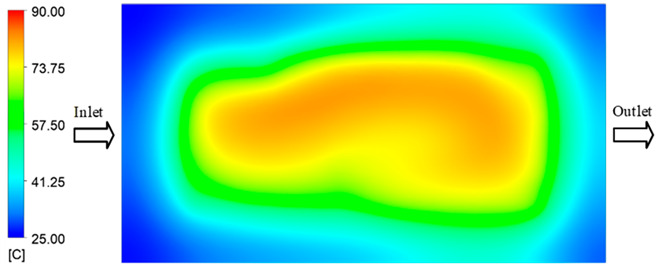	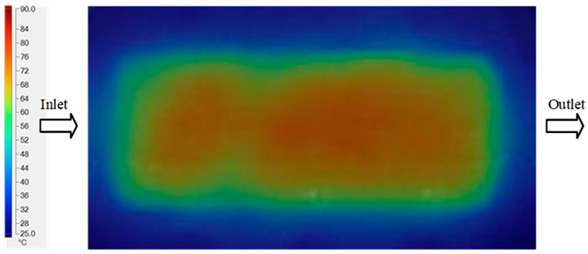
12	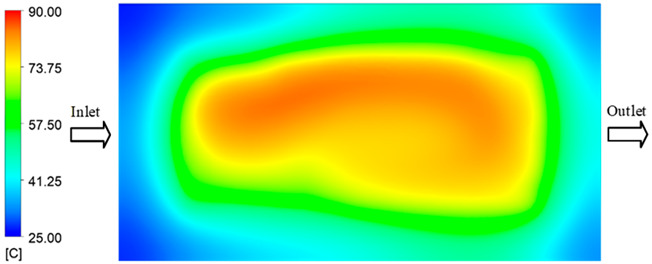	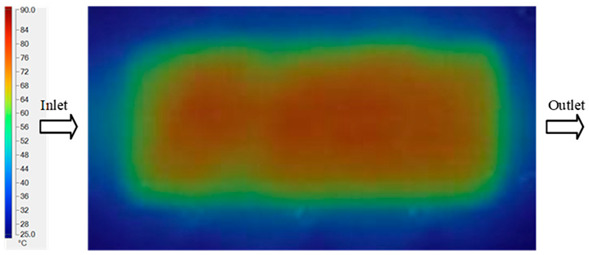
14	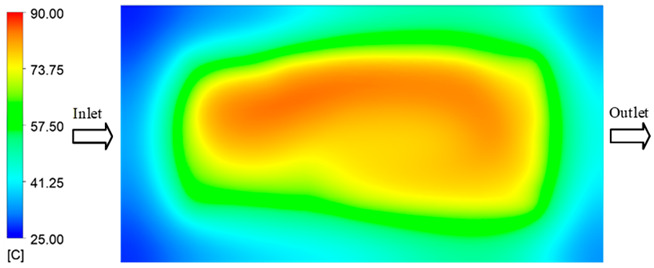	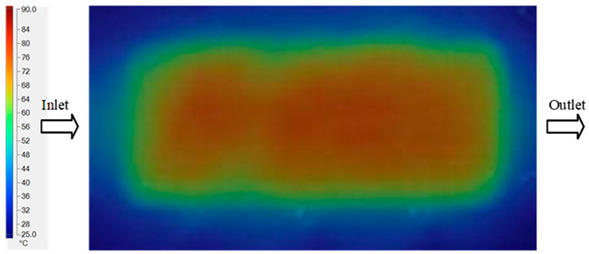
16	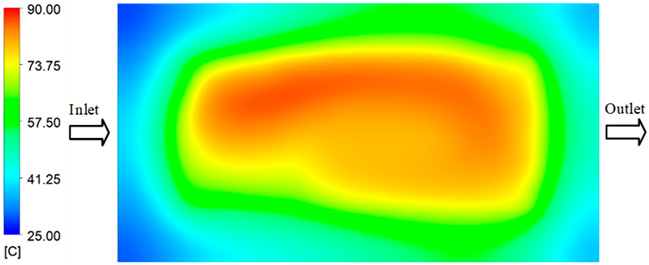	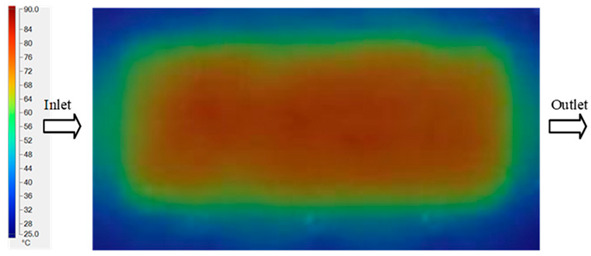
18	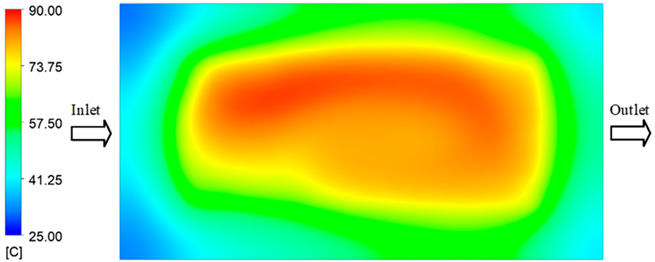	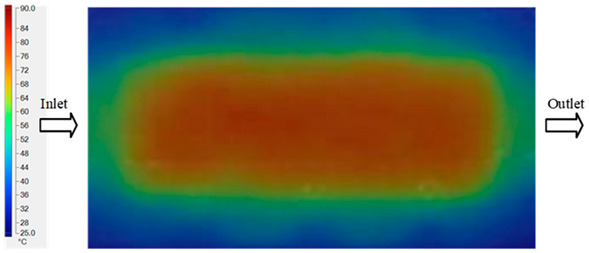
20	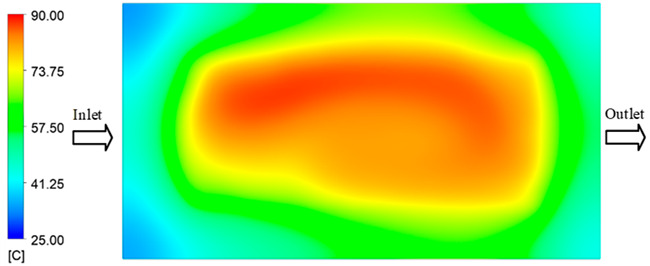	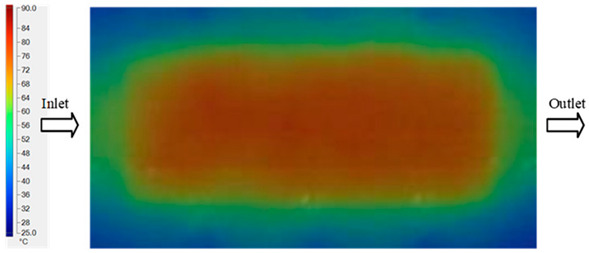

**Table 4:** Maximum temperature values from simulation and experiment at different inlet temperatures (70 °C, 80 °C, 90 °C).

Time (s)	Maximum Temperatures from the Graph					
	Simulation (70 °C)	Experiment (70 °C)	Simulation (80 °C)	Experiment (80 °C)	Simulation (90 °C)	Experiment (90 °C)
2	45.6	43.1	50.4	47.3	53.4	51.2
4	54.4	51.1	59.1	56.6	63.6	59.1
6	55.6	53.6	64.8	61.8	70.4	68
8	59	56	67.3	64.7	73.2	72.1
10	61.8	59	68.2	66.7	75.8	74.8
12	63.7	60.2	68.5	68	79	77.5
14	65.1	61.1	69.3	68.7	78.7	77.8
16	64.2	60.6	70	69.9	79.1	78.6
18	64.4	61.3	70.5	70.2	79.5	78.5
20	65	61.3	70.8	70.5	79.8	79.2

**Table 5:** Thermal–physical properties of Polypropylene (PP).

Density (g/cm^3^)	Specific Heat (J/(kg·K))	Melting Temperature (°C)	Molding Shrinkage (%)	Melt Flow Index (g/10 min)	Coefficient of Thermal Expansion (10^−6^/K)
0.90–0.91	1900–2300	160–170	1.0–2.5	2–30	100–180

**Table 6:** Typical injection molding parameters for Polypropylene (PP).

Melting Temperature (°C)	Barrel Temperature (°C)	Nozzle Temperature (°C)	Mold Temperature (°C)	Holding Time (s)	Holding Pressure (MPa)	Cooling Time (s)	Screw Speed (rpm)
160–170	180–240	180–230	20–60	3–12	30–80	10–30	30–80

**Table 7:** Simulation and experimental filling of the Partial tooth mold under different injection pressures.

Injection Pressure (MPa)	Simulation	Experiment
3	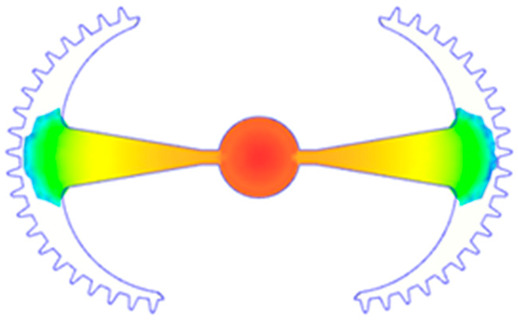	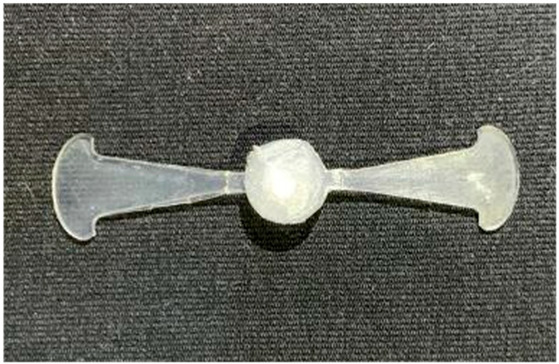
5	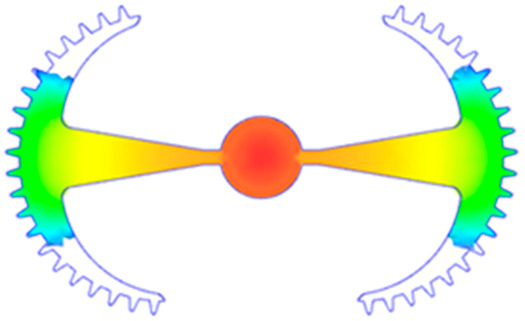	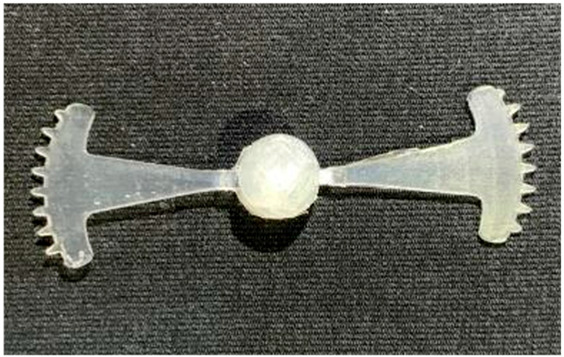
6	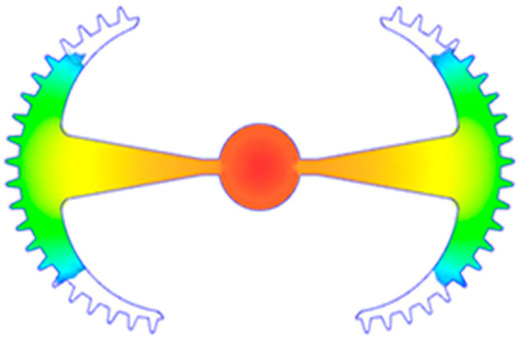	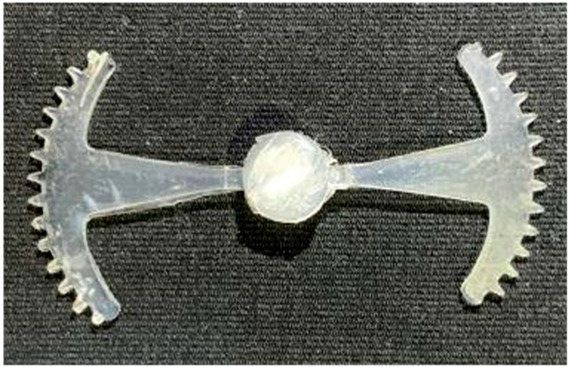
10	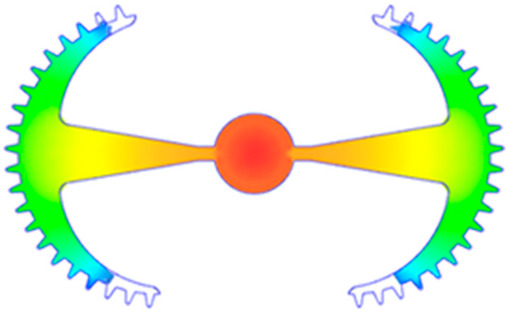	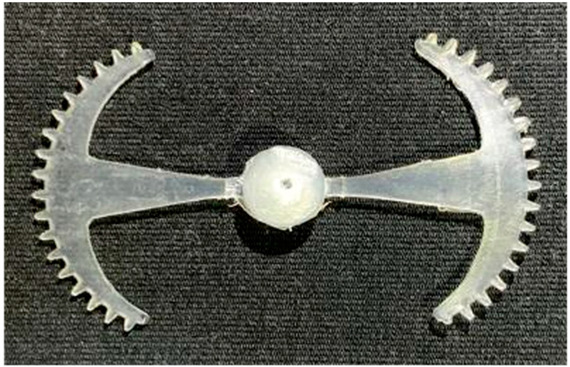
12	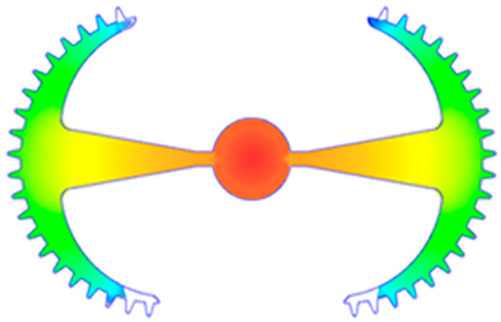	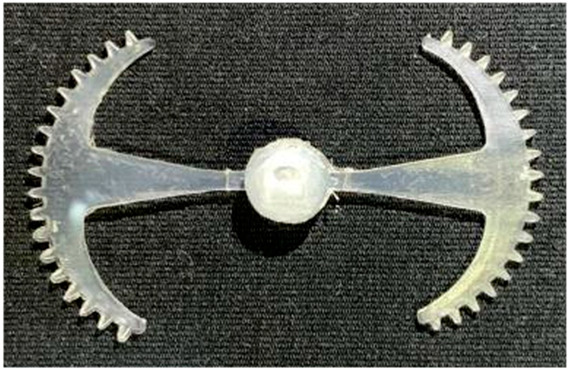
13	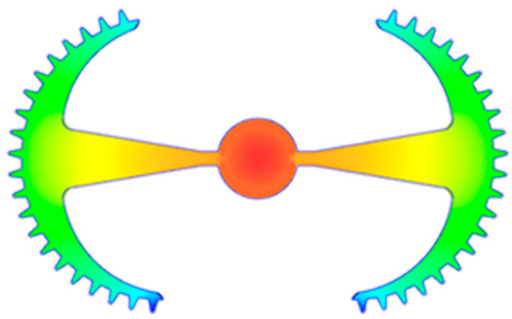	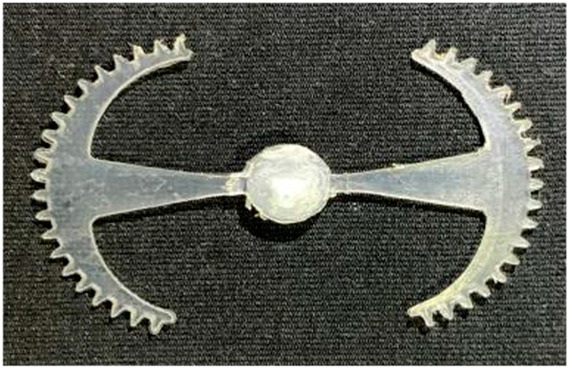

**Table 8:** Comparison of simulation and experimental fill volume at different injection pressures for the Partial tooth mold.

Injection Pressure (MPa)	Simulation Fill Volume (mm^3^)	Simulation (%)	Experiment Fill Volume (mm^3^)	Experiment (%)
3	253.294	61.80	254.470	62.09
5	329.461	80.38	312.987	76.36
6	375.478	91.61	356.302	86.93
10	397.147	96.90	383.183	93.49
12	395.301	96.45	394.450	96.24
13	409.861	100	409.861	100

**Table 9:** Simulation and experimental filling of the hook-shaped leaf mold under different injection pressures.

Injection Pressure (MPa)	Simulation	Experiment
5	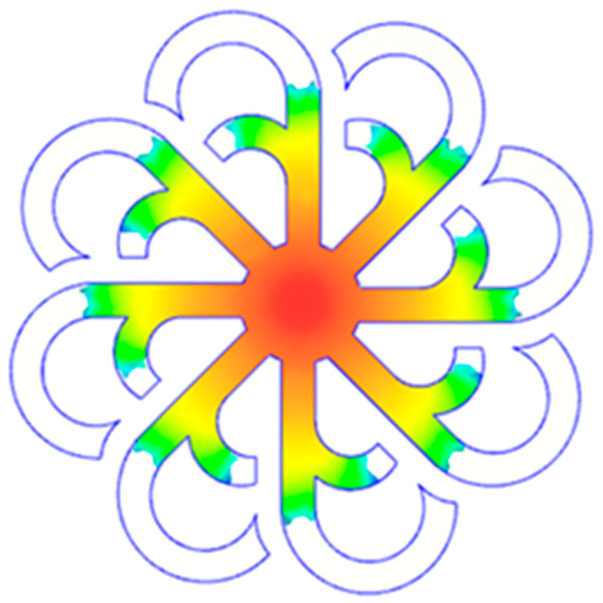	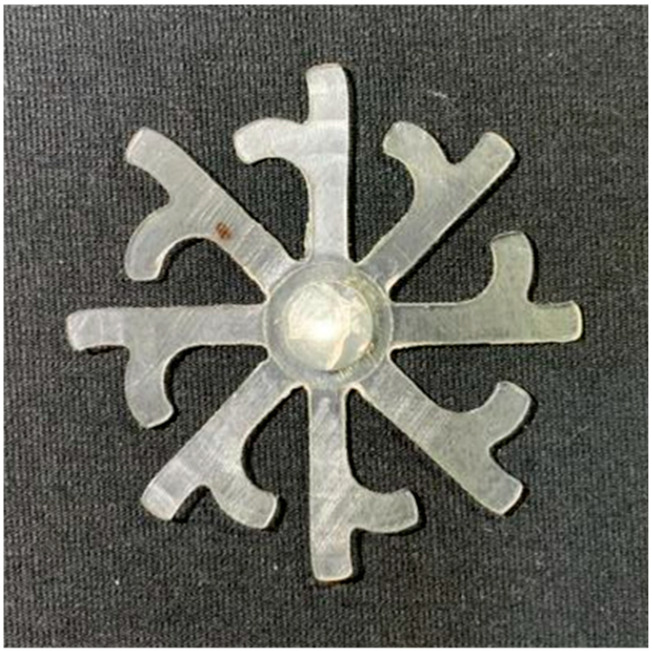
6	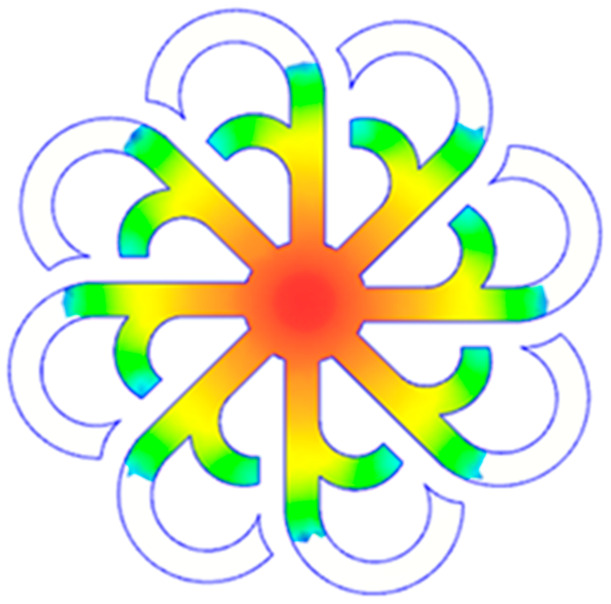	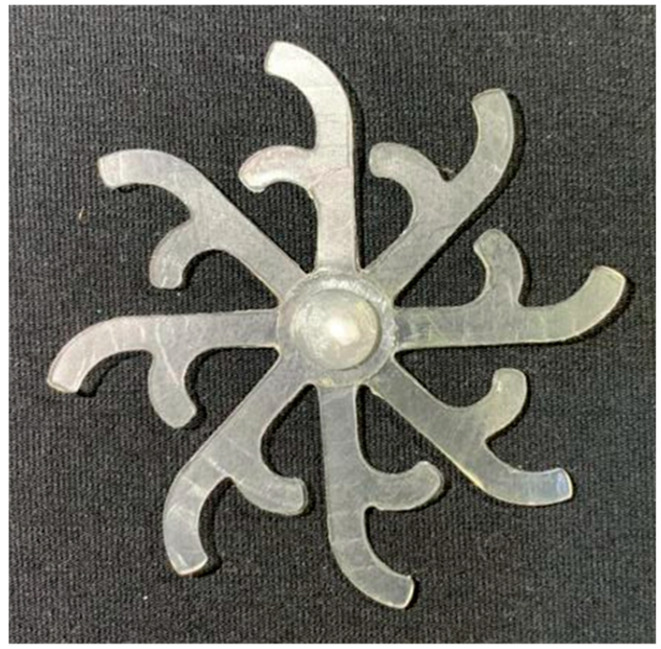
10	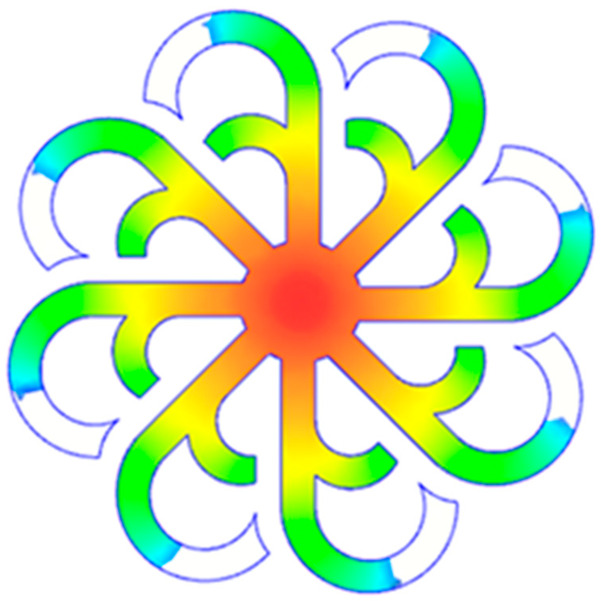	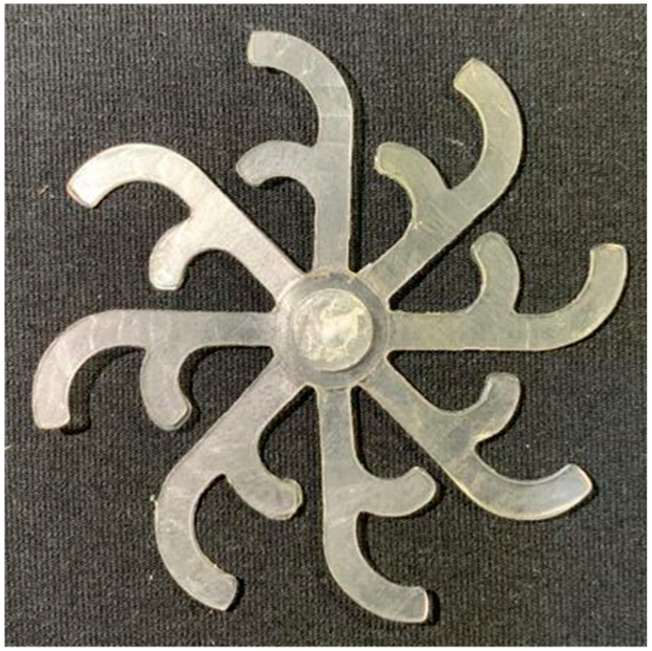
13	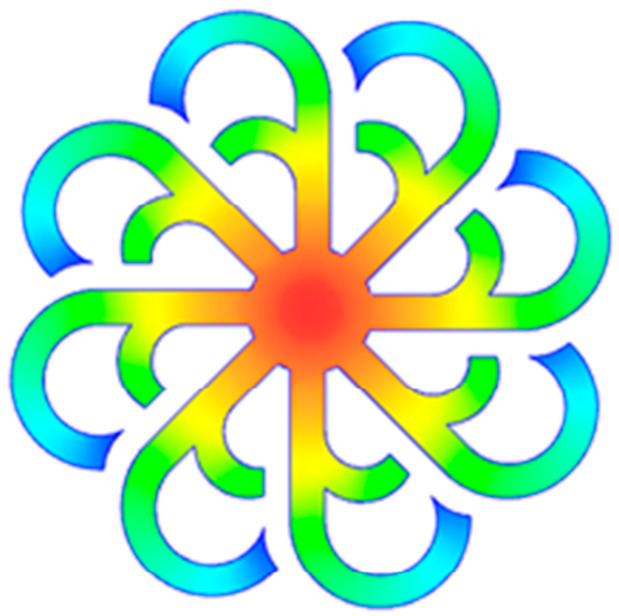	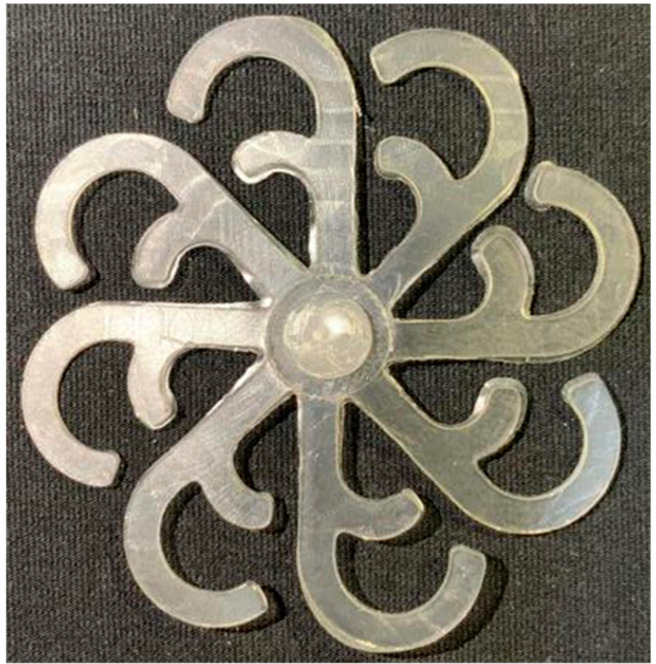

**Table 10:** Comparison of simulation and experimental fill volume at different injection pressures for the hook-shaped leaf mold.

Injection Pressure (MPa)	Simulation Fill Volume (mm^3^)	Simulation (%)	Experiment Fill Volume (mm^3^)	Experiment (%)
5	748.545	53.42	710.550	50.71
6	967.413	69.04	935.576	66.77
10	1145.193	81.73	1134.963	81.00
13	1401.140	100	1401.140	100

## Data Availability

The data presented in this study are available on request from the corresponding author. The data are not publicly available due to proprietary restrictions and ongoing related research.
